# Yield of community-based tuberculosis targeted testing and treatment in foreign-born populations in the United States: A systematic review

**DOI:** 10.1371/journal.pone.0180707

**Published:** 2017-08-07

**Authors:** Mohsen Malekinejad, Andrea Parriott, Amanda P. Viitanen, Hacsi Horvath, Suzanne M. Marks, James G. Kahn

**Affiliations:** 1 Phillip R. Lee Institute for Health Policy Studies, University of California, San Francisco, San Francisco, California, United States of America; 2 Global Health Sciences, University of California, San Francisco, San Francisco, California, United States of America; 3 The Consortium for the Assessment of Prevention Economics (CAPE), University of California, San Francisco, San Francisco, California, United States of America; 4 Centers for Disease Control and Prevention, Division of Tuberculosis Elimination, Atlanta, Georgia, United States of America; McGill University, CANADA

## Abstract

**Objective:**

To synthesize outputs and outcomes of community-based tuberculosis targeted testing and treatment (TTT) programs in foreign-born populations (FBP) in the United States (US).

**Methods:**

We systematically searched five bibliographic databases and other key resources. Two reviewers independently applied eligibility criteria to screen citations and extracted data from included studies. We excluded studies that contained <50% FBP participants or that examined steps only after diagnosis of latent TB infection (LTBI). We stratified studies as majority FBP (50–90%) and predominantly FBP (>90%). We used random-effects meta-analytic models to calculate pooled proportions and 95% confidence intervals (CI) for community-based TTT cascade steps (e.g., recruited, tested and treated), and used them to create two hypothetical cascades for 100 individuals.

**Results:**

Fifteen studies conducted in 10 US states met inclusion criteria. Studies were heterogeneous in recruitment strategies and mostly recruited participants born in Latin America. Of 100 hypothetical participants (predominantly FBP) reached by community-based TTT, 40.4 (95% CI 28.6 to 50.1) would have valid test results, 15.7 (95% CI 9.9 to 21.8) would test positive, and 3.6 (95% CI 1.4 to 6.0) would complete LTBI treatment. Likewise, of 100 hypothetical participants (majority FBP) reached, 77.9 (95% CI 54.0 to 92.1) would have valid test results, 26.5 (95% CI 18.0 to 33.5) would test positive, and 5.4 (95% CI 2.1 to 9.0) would complete LTBI treatment. Of those with valid test results, pooled proportions of LTBI test positive for predominantly FBP and majority FBP were 38.9% (95% CI 28.6 to 49.8) and 34.3% (95% CI 29.3 to 39.5), respectively.

**Conclusions:**

We observed high attrition throughout the care cascade in FBP participating in LTBI community-based TTT studies. Few studies included cascade steps prior to LTBI diagnosis, limiting our review findings. Moreover, Asia-born populations in the US are substantially underrepresented in the FBP community-based TTT literature.

## Introduction

Undiagnosed latent tuberculosis infection (LTBI) is present in nearly one-third of the world population. LTBI prevalence is relatively low (point estimates of 4.7% and 5.0% in the overall population of the United States; however, point estimates are 15.9% and 20.5% in foreign born populations (FBPs) in the United States [1], with point estimate variation within each population due to use of different diagnostic tests. Those with untreated LTBI are at heightened lifetime risk (5–10%) of developing tuberculosis (TB) disease [2] and the risk substantially increases in the presence of risk factors such as smoking (2.5 times) [3], and co-infection with human immunodeficiency virus (HIV) (10 times) [2]. Other medical risk factors such as diabetes, substance use, being on immunosuppressive medications, and other conditions can also increase this risk [4].

In the United States, FBP are at high risk of both LTBI and TB disease, with wide variation by country of origin [5,6]. FBP comprised 66.5% of TB disease cases in the United States in 2014 [5] with a case rate more than 13 times higher than that of US-born individuals [5]. From 2010 to 2014, five nations represented 54% of FBP with TB disease: Mexico (21%), the Philippines (12%) Vietnam (8%) India (8%) and China (7%) [5].

Testing for TB disease is required for persons seeking permanent residence in the United States (i.e., immigrants and refugees), but it is not routinely required for nonimmigrants that are issued temporary visas (e.g., students and skilled workers). Despite being at high risk for LTBI, FBP entering the United States, regardless of their immigration status, are not required to complete LTBI treatment to prevent development of TB disease [5]. Further, “undocumented” FBP are at heightened risk of developing TB disease because they are less likely to be tested and treated for LTBI [5] and more likely to have undiagnosed TB disease due to lack of health insurance or fear of deportation [7]. Thus, identifying and treating LTBI in FBP, including the undocumented, is essential to eliminating TB disease in the United States [8]. Strategies for identifying “hidden” FBP, including undocumented migrants and immigrants can require special effort, as groups may be isolated from the general population [9].

In contrast to “passive” identification in which the healthcare system relies on providers to identify and treat persons with LTBI and TB, or refer persons with LTBI or suspected TB to public health departments for further evaluation and treatment, “active” approaches proactively identify and screen populations at high risk for disease [10]. There are four such approaches in the United States: 1) contact investigation: identification and screening of individuals who came into contact with a person with infectious TB disease [11]; 2) outbreak investigations, where members of a community undergoing a TB outbreak are targeted for testing without attempting to ascertain contact with individual infectious cases [12]; 3) institution-based: routine screening of individuals at risk for TB before entry into an institution or during their residence (e.g., shelters, nursing homes) [13,14] and mandatory screening in correctional settings [15]; and 4) community-based: targeted identification, testing, and treatment of individuals at risk for TB in community settings (e.g., streets, social gatherings, residential areas) [16,17]. Although all these approaches are important for increasing LTBI testing in the United States, targeted community-based strategies may reach certain subpopulations of FBP that might not be reached using other approaches.

We systematically reviewed community-based TB targeted testing and treatment (TTT) programs targeting FBP in the United States to estimate their yield and efficiency, evaluating proportions of participants retained at each step of the test and treatment cascade of care (e.g., participants recruited, tested, and received treatment). We also explored which recruitment methods may yield higher retention in each step of the community-based TTT programs.

## Methods

**Fig 1:**
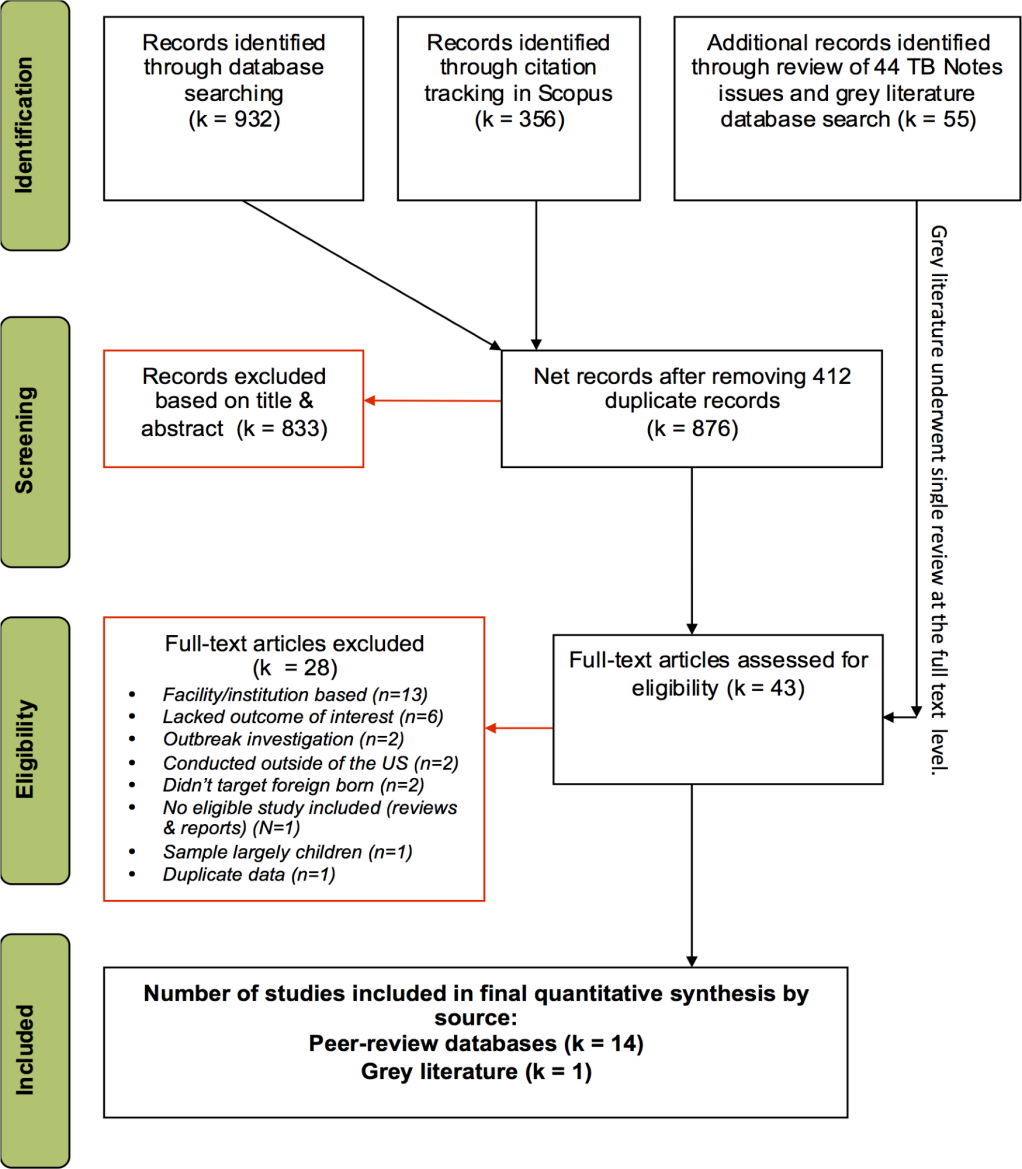
PRISMA flowchart depicting screening process.

We based our methods on those of the Cochrane Collaboration [18], developed a systematic review protocol in advance (S1 File) and reported findings according to the Preferred Reporting Items for Systematic Reviews and Meta-Analyses (PRISMA) recommendations (see **Fig 1** for flow diagram and **S2 File** for PRISMA checklist) [19].

### Eligibility criteria

Our review focused on studies that recruited adult FBP for targeted TB testing and treatment in community settings in the United States. All quantitative study designs (e.g., randomized or non-randomized controlled trials, cohort, cross-sectional, and other observational studies) were eligible for inclusion. We excluded studies that were primarily qualitative in nature, as well as those focused on diagnostic test accuracy. We used the population, intervention (strategy), comparator, and outcome (PICO) schema to further detail the eligibility criteria as outlined below [18].

#### Population

A study was eligible if the FBP comprised at least 50% of total participants, or per contextual information provided by the study, it was assumed that the majority (50%-90%) of participants were FBP, or the output and outcome data were reported predominantly (>90%) for FBP.

#### Identification and recruitment strategies and comparator

We included studies in which investigators made initial contacts to identify FBP for LTBI testing in community settings, including camps, streets, churches, social events, homes and community centers and other public spaces. Eligible recruitment strategies were those aiming to (a) increase the likelihood or expedite the process of recruiting participants for LTBI testing, and/or (b) increase the likelihood or expedite the process of accepting TB testing. Presence of a distinct comparator group was not a requirement. We excluded institutions-based strategies (i.e., using pre-existing lists of names with personally-identifiable demographic and contact information to identify FBP in schools, jails, prisons, detention centers or hospitals). We did not include studies that primarily focused on strategies to retain participants in treatment.

#### Outputs and outcomes

Primary metrics included the frequency of participants for all steps in the community-based TTT cascade including number of individuals, a) who were reached; b) recruited into the program; c) with tuberculin skin tests (TST) placed or specimens collected for Interferon Gamma Release Assay (IGRA); d) with valid test results; e) testing positive; f) who received chest radiograph (CXR) to rule out active TB disease; and g) who were offered; h) who started; and i) who completed LTBI treatment. We defined “reached” as: individuals from the target population as having been initially contacted; “recruited” as: individuals eligible and agreed to receive TB testing; “tested” as: individuals with a TST placed or who underwent blood draws for IGRA; “with valid test results” as: individuals returning 48–72 hours after TST placement to have their TST read or with a confirmed laboratory result for IGRA; “tested positive” as defined by studies; and “received a CXR” for examination of TB disease. S1 File provides more detail.

### Search methods

We conducted a comprehensive search for peer-reviewed articles and the “grey literature” in an effort to identify all relevant studies. Studies could be published, unpublished, in press and in progress, indexed at any date, and written in any language.

#### Electronic search

We developed our search strategy using combinations of relevant Medical Subject Heading (MeSH) terms and appropriate keywords in PubMed (**S3 File**). We adapted this strategy for use in the other bibliographic databases (Web of Science, Scopus, PsycINFO and the Cochrane Central Register of Controlled Trials). We completed our searches in March 30, 2015. Using advanced syntax in Google, we also searched the web sites of US state health departments as well as those of major US cities and counties. We searched doctoral dissertations using ProQuest Dissertations as well as all available conference abstracts (2007–2015) from the American Public Health Association. We used Scopus to identify additional studies cited by studies included in our review or citing them.

#### Other sources

We contacted authors (first, second, and last) of our included studies and other experts to learn of any relevant ongoing studies or that may be in preparation or in press. Additionally, we thoroughly hand-searched 44 issues of TB Notes (a quarterly newsletter published by CDC containing programmatic updates and which lists new CDC TB publications) [20] and other grey literature documents identified through targeted searching.

### Methods for selection of studies

One author (HH) performed a broad first cut of all titles from the electronic searches to exclude citations that were obviously irrelevant. Two authors (AP, AV) then screened the titles, abstracts and descriptor terms of the retrieved citations to identify potentially eligible studies according to the pre-specified inclusion criteria. They then independently reviewed the full text of all potentially eligible citations. Any discordance in opinion of the two reviewers as to whether or not a study met our inclusion criteria was resolved by discussion with a third arbiter (MM).

### Data extraction and management

Two authors (AV, AP) extracted data into a standardized, pre-piloted data extraction form (**S4 File**). Several key characteristics were extracted from each study: citation, study design characteristics of the population and study setting, recruitment methods, outputs, and outcomes. We defined populations as being targeted if the larger population in a defined geographical setting was perceived by investigators as being at high risk for TB or LTBI but beyond the program’s immediate access for testing. For outputs, we extracted the number of participants reported at stages throughout the community-based TTT cascade: reached, recruited, tested, had valid test results, tested positive, received a CXR for TB disease, were offered LTBI treatment, started on LTBI treatment, and completed LTBI treatment. Contextual data (e.g., study setting, target population) were extracted by one of the authors of this study and then checked for accuracy by another author. All output data were extracted independently by two authors, and then reconciled.

### Statistical analysis and data synthesis

We created tables in Excel [21] to organize and synthesize data and conducted descriptive analyses of included study characteristics. We categorized extracted data into two groups based on the proportion of the sample that were: predominately FBP if >90% were FB, and majority FBP if 50%-90% were FBP. We stratified data analysis this way because LTBI prevalence was expected to vary substantially by the proportion of FBP among participants. While some studies focused almost exclusively on recruiting FBP, others recruited participants from work or community settings where high density of FBP was expected, but not the entire sample turned out to be FBP. Further, we intended to explore the potential effect of the composition of sample in respect to percent of FBP on program performance. We calculated pooled proportions of participants retained in the community-based TTT cascade steps with inverse variance-weighted random-effects meta-analytic models using Stata with the metaprop module, which calculates pooled estimates of proportions, and the ftt option, which transforms individual study proportions prior to pooling using the Freeman-Tukey double arcsine transformation [22–24]. We also conducted sub-analyses for these pooled proportions by recruitment and retention strategies.

In order to calculate the cumulative proportion of participants retained in each cascade step (from the number of persons initially reached), we used the proportion of participants retained between adjacent steps of the cascade. The cumulative proportions are based on the products of the proportions retained in adjacent cascade steps (e.g., the proportion tested of those reached is equal to the product of the proportion recruited of persons reached and the proportion tested of persons recruited). To calculate 95% confidence intervals for the cumulative proportions that accounted for cumulative uncertainty in a previous cascade step, we used a simulation method. First, the pooled proportion estimate and the confidence limit closest to 0.5 were transformed using the Freeman-Tukey double arcsine transformation, using the harmonic mean of the individual sample sizes as the sample size and the harmonic mean times the pooled proportion as the number of successes. We then took 50,000 draws for each adjacent step from a normal distribution with mean equal to the transformed pooled proportion and standard deviation equal to the difference between the transformed pooled proportion and transformed confidence limit divided by 1.96. Draws were then reverse transformed into proportions, and multiplied together to form a probability distribution for the cumulative proportions, and the 95% confidence limits were taken from the 2.5th and 97.5th percentile of these distributions. Because normal distributions have no upper or lower boundaries, we replaced draws that were less than the transformed value of zero or more than the transformed value of one with the transformed values of zero and one, respectively. In the special case where the pooled probability was equal to one, we used a truncated normal distribution, taking draws only from the half of the distribution that was less than the transformed value of one.

## Results

**Fig 1** illustrates our screening process. **S5 File** provides references for all records examined at full-text level. Fifteen studies met inclusion criteria.

From 15 included studies (**Table 1**), seven studies reported data only on P-PFB (>90%) [16, 25–30]; four reported data only on majority FBP (50%-90%) [31–34] and four reported on both [35–38]. Studies that met inclusion criteria took place between 1986 and 2012 in 10 states, including California, Connecticut, Delaware, Florida, Indiana, Maryland, Minnesota, New York, North Carolina, and Virginia. Eleven were conducted in rural areas, three in urban areas, and one in both. Eight studies (53%) targeted farmworker populations [28,29,31–35,37]. **S4 File** provides more detail.

**Table 1:** Characteristics of 15 community-based TB targeted testing and treatment studies among foreign-born populations in the United States.

Study	Target population (study setting)	Identification method	Recruitment start date (recruitment duration)	Demographic characteristics	Recruitment and retention strategies	Participants reached/number with valid test	Tx regimen and completion rates in those starting tx
**Studies that reported data for predominantly (>90%) foreign born populations alone**							
[28]	Mexican and Mexican-American migrant farm workers, Orleans and Monroe counties, New York (rural)	Residence-based (farmworker camps)	May 1997 (4–5 months)	Sex: 79.6% male; Age: Median 29 years (SD 10.7)	Peer migrant bilingual/bicultural staff, project activities in the migrant camps (except CXR); Spanish materials	206/149	NR/NR
[16]	Immigrant Latin Americans, Baltimore, Maryland (urban)	Venue based (churches & community centers, service agencies, restaurants)	1997 (NR)	Origin: Latin American countries	Bilingual staff, Spanish materials, partnership with Hispanic serving businesses, churches, and agencies	NR/136	9 months INH/100%
[26]	Immigrant taxi drivers, New York, New York (urban)	Workplace-based (Social fair in airport)	July 2002 (1 day)	Sex: 100% male; Origin: 25 different countries; Age: all >19 years	Multilingual staff, placing and reading tests at an easily accessible location with flexible schedules, short visits, phone calls made to those who didn’t return for the test reading on day one	NR/78	9 months INH/50%
[27]	Persons born in high TB prevalence countries living in Suffolk County, New York	Venue-based (churches, adult education centers, community social services—food and clothing pantries)	2000 (3–4 years)	NR	Multilingual staff, involvement of community leaders, testing in various community settings, educational sessions provided, education materials (pamphlets and CDs) in multiple languages, onsite CXR machine.	3310/1303	4 months Rifampin/ 77.9%
[29]	Migrant farmworkers, Connecticut (rural)	Residence-based (farmworker barracks)	2005, growing season (NR)	Sex: 100% male; Origin: 96% Mexico; Age: 16–59 years	Bilingual staff, convenient (on-site) testing location, outreach, education	130/57	9 months INH/NR ^1^
[30]	Immigrants & refugees, Rochester, Minnesota (urban)	Venue-based (adult education center)	March 2009 (1–2 months)	Sex: 59.8% male; Origin: 39% African, 30% Latin American, 21% Asian, 8% Middle Eastern, <1% other; Age: all >18 years	Used community based participatory research to design screening strategy	584/259	9 months INH/63.9% ^2^
[25]	FB Latin Americans, Baltimore, Maryland (urban)	Venue-based (community health center & social fair)	2006 (4 years)	Sex: 63.5% male; Origin: 14 different Latin countries; Age: all >18 years, median 34 (SD 10.8)	Bilingual staff, Spanish materials, partnering with Hispanic serving CBOs	NR/391	NR/63%.
**Studies that reported data for majority (50–90%) foreign born populations alone**							
[31]^3^	Migrant farmworkers, coastal Delaware, Maryland, Virginia (rural)	Residence-based (migrant camps)	June 1984-June 1985 (two growing seasons)	Origin: Most FB Haitian; Age: 18% <15 years (Eastern Shore participants only)	Testing offered at convenient location& time (after work).	NR/1263	INH/NR
[34]	Hispanic migrants, Surry County, North Carolina (rural)	Workplace-based & venue-based (social gathering areas)	1 Jul 1988 (92 days)	Sex: 89% male; Age: 17% <20 years	Testing in workplaces and social gathering areas, bilingual pamphlet, testing explained by interpreter or public health nurse.	461/435	INH/NR
[32]	Migrant farm workers, Immolakee, Florida (rural)	Residence-based (migrant camps)	1 Feb 1992, (56 days)	Sex: 80% male; Origin: 53% Hispanic, 42% black non-Hispanic; Age: >16 years	Testing conducted in camps after working hours	518/267	9 months INH/NR
[33]^3^	Migrant farm workers, Orleans and Monroe counties, New York (rural)	Residence-based (farmworker camps)	Jun 1994-Jun 1995 (6 months)	Origin: Hispanic (1994: 96%, 1995:99%); Age: >20 years	Bilingual staff, Outreach team from community, testing at migrant camp, provided education about TB and testing, advertised screening in Spanish	NR/371	6 months INH/26.62% (≥6 months); 2% entire course
**Studies that reported data on both majority and predominantly foreign-born populations**							
[36]	Migrant farmworkers, coastal Delaware, Maryland, Virginia (rural)	Residence-based (farmworker camps)	25 Jul 1982 (~6 weeks)	*M-FBP*: Sex: 67.5% male; Origin: Haiti, Mexico, other; Age: all >5 years (5–14 years: 8%). *P-FBP*: Sex: 73% male; Origin: 34% Haitian, 31% US blacks, 18% US born Latinos 14% Mexican born Latinos; Age: all >5 (5–14 years: 8%).	Study in convenient location	M-FBP: NR/338; P-FBP: 855-902/709	NR/NR
[38]	Latinos, San Francisco, California (urban)	Advertisement	Aug 1983 (7–8 months2)	*M-FBP*: Sex: 44% male; Age: mean 22.4, 34% <10, 18% (10–19). *P-FBP*: Origin: 36% El Salvador, 32% Mexico, 16% Nicaragua, 6% Guatemala, 10% other; Age: all >20 years	Latino staff, highly publicizing screening, screening located within primary Latino community	M-FBP: NR/1871; P-FBP: NR/794	9 months INH/NR
[35]	Migrant farmworkers, Indiana (rural)	Residence-based (farmworker camps)	Jul 1991 (3 months)	*M-FBP*: Origin: Mexico; Age: all >11 years. *P-FBP*: Sex: 63.3% male; Origin: 97.9% Hispanic; Age: all >18 yr, Median 27 (range 18–69).	Testing offered at convenient location& time (after work)	M-FBP: 595/318; P-FBP: 595/157	6 months INH/64% (<2 months) and 9% (6 months)
[37]	Hispanic migrant farmworkers, Yolo County, California (rural)	Residence-based (farmworker camps)	Jul 1995 (1–3 months^4^)	*M-FBP*: Sex: 43.5% male Origin: Hispanic and Haitian; Age: Median 27.9 years (SD 17.1); 6.1% <15 years. *P-FBP*: Origin: All but one from Mexico.	Bilingual staff, flyers advertising the health fair, mobile CXR clinic	M-FBP: 669/269; P-FBP: 669/237	6 months INH/90%

Legend: CBOs, Community-based organizations; INH, Isoniazid; M-FBP, majority (50–90%) foreign born population; NR, not reported; P-FBP, predominantly (>90%) foreign born population; tx: treatment

Footnotes

1. No-one followed up with the community health center to get prescribed LTBI treatment

2. Of those offered treatment

3. Percent foreign born not stated; assumed at minimum M-FBP due to migrant farmworker status

4. Exact value unclear

With respect to methods used to identify participants, eight (53%) studies were residence-based [28,29,31–33,35–37]. For instance, McCurdy 2012 [37] recruited Spanish-language migrant farmworkers from two camps in Yolo County, California. Two studies targeted persons at the work-place (one combined with other venues) [26,34]. Gany 2005 [26] recruited taxi drivers at high risk for TB in New York City by holding a one-day social fair at an airport and offering a convenient location and methods for reading the TST results. Perez-Stable 1986 [38] was the only advertisement-based study that solely used social marketing recruitment strategies and offered multiple conveniently located testing sites in Latino community in San Francisco, California [38]. Five studies conducted recruitment at other venue types such as community health centers, social fairs, churches, and community centers. [16,25–27,30]. For example, Wieland 2011 [30] contacted FBP through an adult education center in Rochester, Minnesota, catering to FBP representing 70 countries.

Most commonly reported recruitment and retention strategies included provision of easily accessible testing sites (14 studies) [16,25,27–38]; use of multilingual staff or interpreters (11 studies) [16,25–29,33–34,36–38]; combining the recruitment and testing steps (10 studies) [16,25–27,29,34–38]; onsite evaluation of participants testing positive for TB infection (six studies) [27,29,31,32,35,37]; and advertising and marketing (five studies) [25,27,33,35,38]. Many studies used multiple strategies to enhance recruitment, for example, combining advertising or marketing with residence based recruitment. All but one study used TST exclusively for screening. Mooney 2006 [27] used a combination of TST and IGRA.

Studies were heterogeneous in terms of reporting of various steps of the community-based TTT cascade data. Eight of the 15 studies did not report the number of persons reached. All studies reported the number of valid and positive tests. The range of number of persons reached was 130–3301 and number of persons with valid test results was 57–1871. Data about number of studies that reported on each step and number of participants in each step can be obtained from **S4 File**.

**Fig 2:**
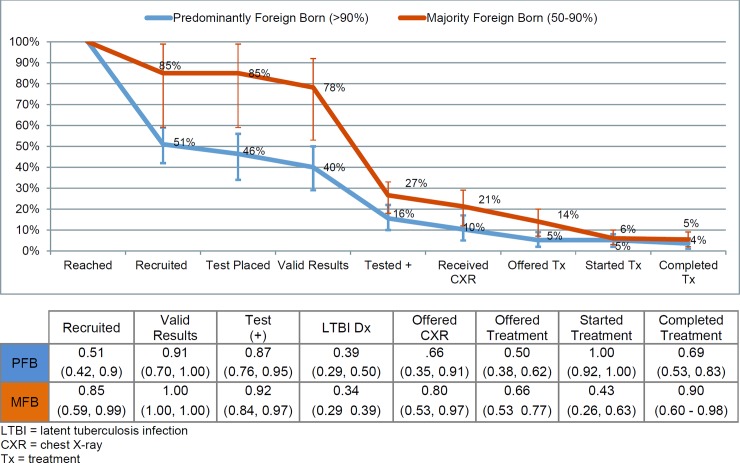
Cumulative proportion of participants retained in subsequent steps once reached via community-based TB testing and treatment in the United States, by percent foreign born.

Based on majority FBP data for the community-based TTT cascade (**Fig 2**), we estimated that in a hypothetical group of 100 participants reached for LTBI testing, 84.7 (95% CI 59.4 to 98.8) would be recruited (and same number tested), 77.9 (95% CI 54.0 to 92.1) would have valid test results, 26.5 (95% CI 18.0 to 33.5) would test positive, 21.2 (95% CI 12.1 to 29.0) would receive a CXR, 14.0 (95% CI 7.6 to 19.7) would be offered treatment, 6.0 (95% CI 2.6 to 10.0) would start treatment, and 5.4 (95% CI 2.1 to 9.0) would complete treatment. Similarly, based on predominantly FBP data, estimates for the cascade steps are as follows: the number who would be recruited 50.9 (95% CI 42.4 to 59.3), tested 46.4 (95%CI 33.8 to 56.1), have valid test results 40.4 (95% CI 28.6 to 50.1), test positive 15.7 (95% CI 9.9 to 21.8), receive a CXR 10.4 (95% CI 4.7 to 16.5), be offered treatment 5.2 (95% CI 2.2 to 8.6), start treatment 5.2 (95% CI 2.2 to 8.4), and complete treatment 3.6 (95% CI 1.4 to 6.0). We presented the pooled proportions for adjacent steps in the cascade under Fig 2. **S6 File** shows proportions in a matric format.

**Fig 3:**
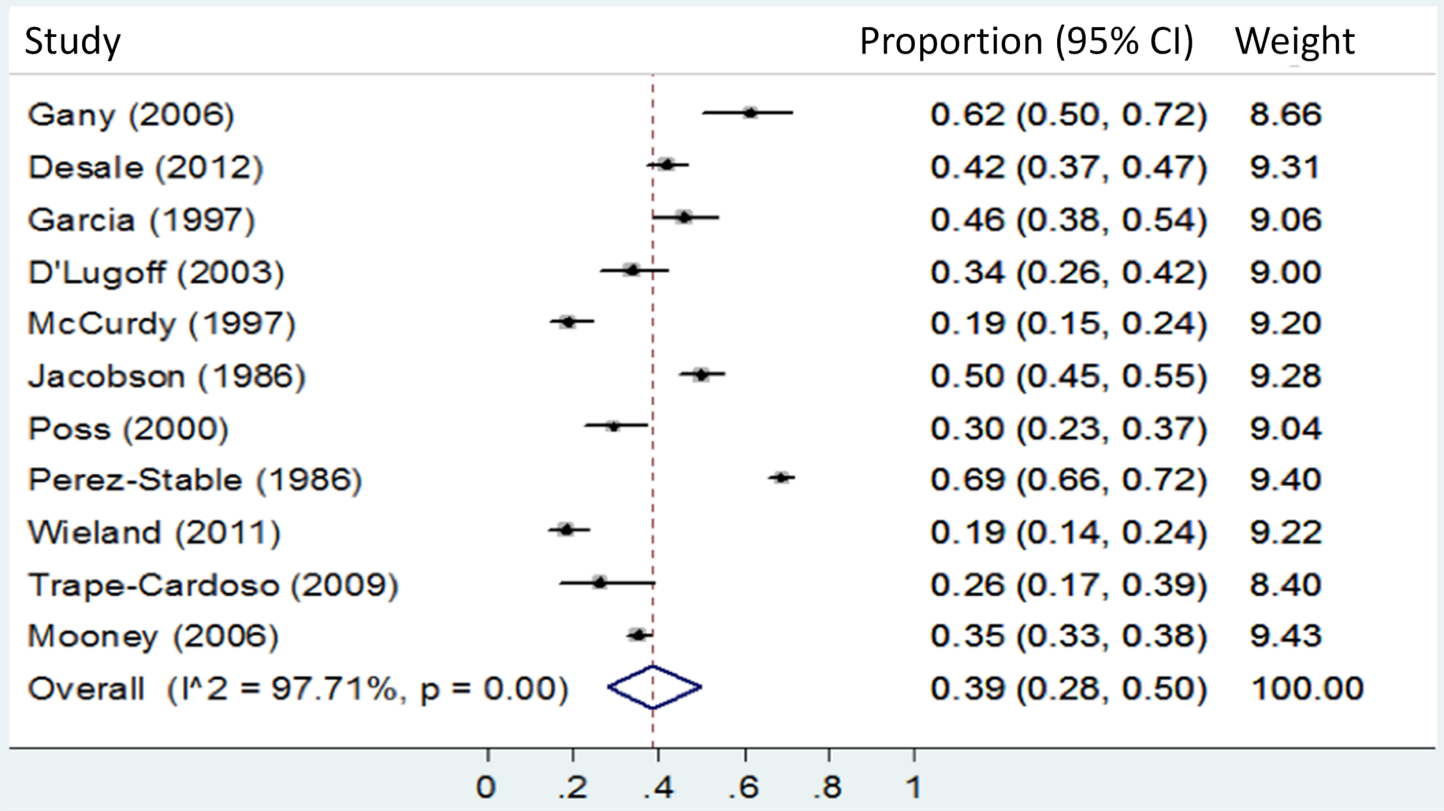
Predominantly (>90%) foreign born populations: Proportion testing positive of those with valid test results.

**Fig 4:**
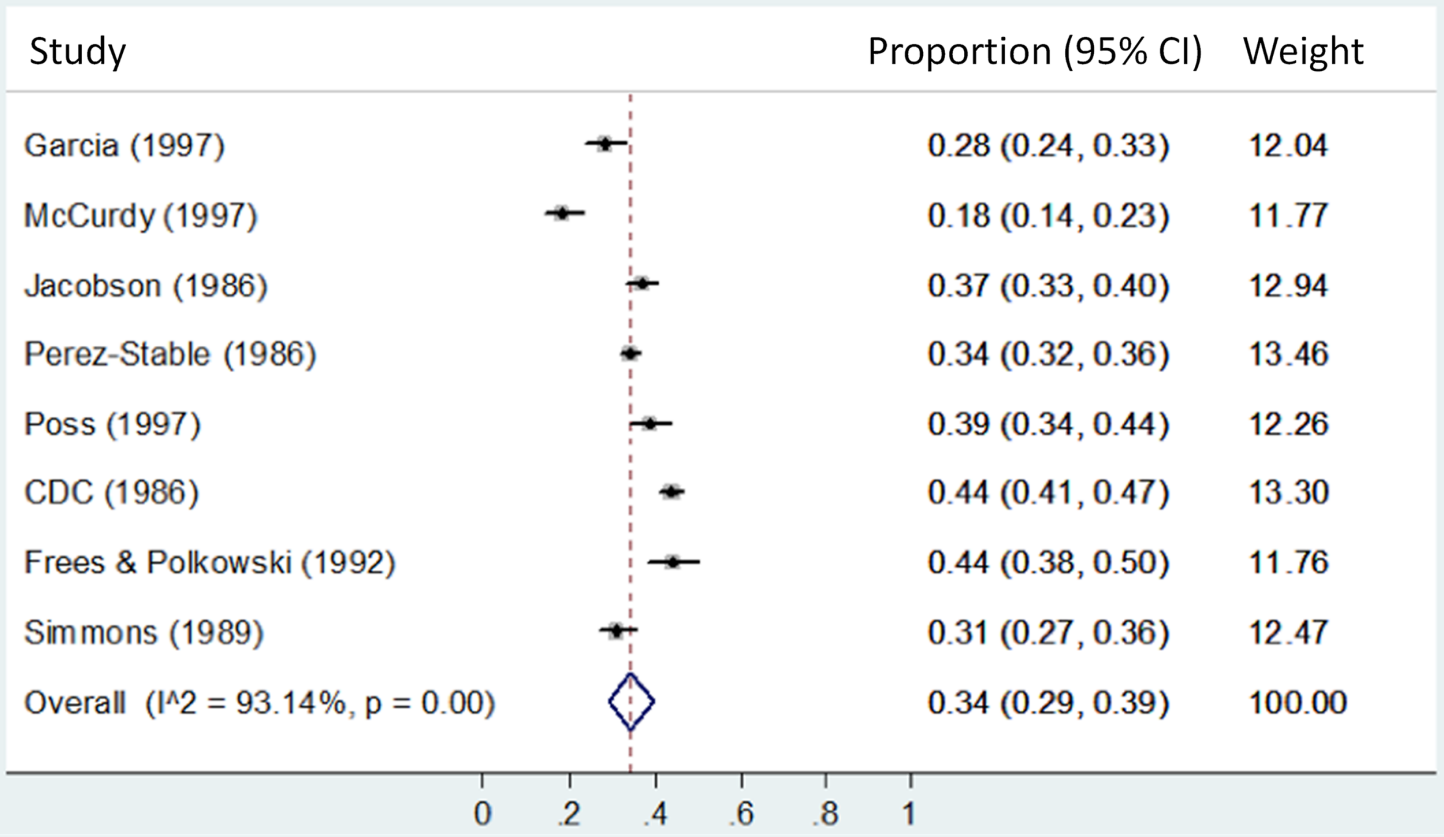
Majority (50%-90%) foreign born populations: Proportion testing positive of those with valid test results.

We present forest plots **(Fig 3** and **Fig 4)** to visually portray the distribution and heterogeneity for the proportion that tested positive of those with valid test results, by percent foreign born: predominantly FBP 39% vs. majority FBP 34%. We observed very substantial (I^2^>93%) statistical heterogeneity among data points included in these analyses.

We did an exploratory analysis to examine whether three implementation approaches of community-based TTT programs (residence-based recruitment, combined recruitment and testing steps, and onsite evaluation of TST) might affect participants’ retention in relevant cascade steps. Overall, the proportion of participants retained was similar across implementation approaches (**Table 2**). We found differences in two scenarios. In studies that provided onsite evaluation of TST for predominantly FBP, of those who were TST+ 97% received a CXR and 82% started treatment, compared with 74% and 42%, respectively, in studies that did not provide onsite evaluation. In studies that provided onsite evaluation of TST for majority FBP, of those who tested positive for TB infection, 68% started treatment, versus 15% for studies without onsite evaluation.

**Table 2:** The TB targeted testing and treatment cascade, by recruitment and retention strategy.

Cascade steps: Describing proportions	Studies	N	Proportion (95% CI)	Studies	N	Proportion (95% CI)
**A**	**Residence-based recruitment**			**Other recruitment strategy**		
**Predominantly FBP**						
Recruited, of those reached	[29, 35]	725	0.46 (0.43–0.50)	[30]	584	0.51 (0.47–0.55)
Received tests, of those recruited	[29, 35]	335	0.98 (0.96–0.99)	[30]	298	0.89 (0.85–0.92)
Received valid results, of those receiving tests	[28, 29, 35]	465	0.92 (0.58–1.00)	[16, 25–27, 30]	2,377	0.84 (0.71–0.94)
**Majority FBP**						
Recruited, of those reached	[35, 36]	1475	0.81 (0.79–0.83)	[36]	461	0.95 (0.93–0.97)
Received tests, of those recruited	[35, 36]	1160	1.00 (1.00–1.00]	[34]	438	1.00 (0.99–1.00
With valid results, of those receiving tests	[35, 36]	1160	0.89 (0.87–0.90)	[34,38]	2631	0.89 (0.88–0.90)
**B**	**Combined recruitment & testing**			**No combined recruitment & testing**		
**Predominantly FBP**						
Recruited, of those reached	[29, 35]	725	0.46 (0.43–0.50)	[30]	584	0.51 (0.47–0.55)
Received tests, of those recruited	[29, 35]	335	0.98 (0.96–0.99)	[30]	298	0.89 (0.85–0.92)
With valid results, of those receiving tests	[16, 25–27, 29, 35]	2,424	0.84 (0.68–0.96)	[28, 30]	418	0.93 (0.91–0.96)
**Majority FBP**						
Recruited, of those reached	[34–36]	1936	0.85 (0.59–0.99)	**—**	0	—
Received tests, of those recruited	[34–36]	1598	1.00 (1.00–1.00)	**—**	0	—
With valid results, of those receiving tests	[34–36]	1598	0.94 (0.93–0.99)	[38]	2193	0.85 (0.84–0.87)
**C**	**Onsite evaluation of TST+**			**No onsite evaluation of TST+**		
**Predominantly FBP**						
Received CXR, of those with (+)	[27, 29]	475	0.97 (0.95–0.99)	[16, 25, 26]	258	0.74 (0.58–0.87)
Offered tx, of those with (+)	[29]	15	0.00 (0.00–0.20)	[16, 26, 30]	142	0.50 (0.18–0.83)
Started tx, of those with (+)	[27, 29]	475	0.82 (0.78–0.86)	[16, 25, 26]	258	0.42 (0.20–0.65)
**Majority FBP**						
Received CXR, of those with (+)	[35, 37]	139	0.90 (0.84–0.94)	[32, 38]	757	0.87 (0.85–0.90)
Offered tx, of those with (+)	[35, 37]	139	0.51 (0.43–0.59)	[34, 38]	774	0.64 (0.61–0.68)
Started tx, of those with (+)	[31, 37]	604	0.68 (0.64–0.72)	[34]	118	0.15 (0.10–0.23)

Legend: CI, confidence interval; CXR, chest x-ray; TST, tuberculin skin test; tx, treatment, (+), positive test result.

Further subpopulation analysis of data by type of target population (migrant farmworker versus others; and majority Latin America-born versus other FB) is provided in **S7 File**.

## Discussion

In this first systematic review of community-based TTT programs in FBP in the United States, 15 studies conducted over 27 years (1986–2012) met our inclusion criteria, seven of which focused exclusively on FBP. Nearly all of the FBP in these studies were from Latin America or the Caribbean. The United States immigrant experience is unique. Hundreds of thousands of persons from high-burden Latin American and Caribbean countries enter the United States each year, many of whom are not authorized to be in the country [39]. There are millions of persons born in Asia or Africa currently residing in the United States who are potentially at elevated TB risk [40], though we found few publications that reported data on these populations [26,27,30].

We extracted data from eligible studies on each step of the TB targeted testing and treatment cascade. Given the prevalence of LTBI in the foreign-born population estimated in the United States by TST of 20.5%, existing community-based TTT programs successfully reached populations at high risk for LTBI: average prevalence of 34–38% (proportion testing positive of those with valid results) depending on the percent FBP in the sample. It is noteworthy that community-based strategies can potentially reach FBP that might not be captured by passive approaches. Passive case finding lacks sensitivity for identification of LTBI in FBP because most providers do not routinely screen FBP for LTBI and if they do, they are not required to report LTBI as a notifiable disease in most U.S. jurisdictions [40]. Further, certain subpopulations of FBP (e.g., seasonal farmworkers, migrants, undocumented individuals) don’t routinely access health care because of language barriers [9], transportation and work schedules issues [26], and fear of deportation. Additionally, migrant FBP may be geographically and culturally isolated and be less likely to seek health care [7], especially if they have low self-perception of TB risk [41]. They often lack health insurance (many are ineligible for federally funded or state programs under current law) and have limited economic resources, and thus are probably less likely to access testing for TB even via active healthcare institution-based LTBI screening approaches.

The publications we found reported on diverse community-based strategies (residence-based, venue-based, work-place based, and advertisement-based), target populations (e.g., farmworkers, taxi drivers, attendee of education center) and settings (urban vs. rural). These programs also used multi-pronged approaches to reach and recruit FBP, including strategies to increase awareness of TB testing, remove language and cultural barriers, accommodate participants travel time and work schedule, and reduce the time burden associated with follow-up visits.

Despite these efforts, we observed substantial attrition in the TB targeted testing and treatment cascade steps among FBP who were reached. The attrition was more profound for predominantly FBP compared to majority FBP particularly in cascade steps preceding “receiving CXR” (e.g., 50% dropped out between reached and recruited for predominantly FBP vs. 15% for majority FBP). One explanation for the observed difference in dropout rates between majority FBP and predominantly FBP is the difference in how testing programs “reached” potential group of participants and the proportion of “true” foreign-born persons among those who were eligible. Predominantly FBP group in our analysis comprised of two types of populations: 1) subpopulations in studies where the overall study sample were majority FBP and studies were unable to ascertain the country of birth before reaching those populations, in which case we used the same number for "reached" for both majority FBP and predominantly FBP, and 2) populations tested through programs that specifically targeted FBP, rather than certain ethnic (e.g., Latino) or occupational group (e.g., farmworker). In the former case, the proportion recruited of reached is always smaller among predominantly FBP, because the number reached is the same for both predominantly FBP and majority FBP, but the number recruited for predominantly FBP is a subset of the number recruited for majority FBP. In the latter case, a large proportion of those "reached" may have declined testing specifically because they were not foreign-born. Thus, the observed difference here says more about what a program can expect by targeting predominantly FBP vs. majority FBP groups. This observed variation may, at least partially, be explained by other factors such as low awareness and lack of knowledge about LTBI among FBP [42], unmeasured factors (e.g., variation in demographic characteristics or recruitment methods), or random variation. Further, findings from a recent study suggest that the rate of treatment completion might even be lower in US-born individuals than FBP [43]. Regardless, better understanding of reasons for attrition can improve efficiencies in future programs. Our exploratory analysis suggests that certain implementation features, such as provision of on-site evaluation of TST results may raise rates of CXR receipt and treatment initiation. However, these differences are across studies; proper assessment would require appropriate within-study controlled comparisons.

Existing community-based TTT published studies that met our study’s inclusion criteria did not focus on reaching Asia-born populations as much as Latinos. FBP coming from certain Asian countries with high prevalence of TB disease (particularly China, India, the Philippines and Vietnam) are at highest risk of LTBI in the United States [5]. It is possible that FBP from Asia receive LTBI screening in other contexts, such as in primary care, but this question fell beyond our review’s scope. It is likewise possible that there was a bias in testing of FBP (toward FBP from Latin American countries) or in publication of studies.

Our findings should be interpreted with caution. First, there was considerable heterogeneity among studies in terms of terminology, definitions and reporting. Most studies did not report on several stages of the cascade, limiting relevant data for analysis. The lack of consistency in reporting explains some of the variations in pooled data.

Second, since all but one study used TST, our findings may thus be less applicable to community-based TTT that will use IGRA in the United States because compared to TST, IGRA has a higher specificity, and it does not require a second patient visit [44]. Thus, rates of FPB receiving test results and potentially starting treatment may be higher with IGRA [45]. This study highlights the need for collecting and reporting of community-based TTT outputs and outcomes for evaluation of IGRA results.

Third, while some included studies reported data on treatment initiation and completion, the main focus of our review (and our search strategy) was to identify studies assessing the yield of various identification and recruitment strategies, and not necessarily strategies for retention in treatment. Our reported retention rates for stages after testing do not represent studies that began from a LTBI diagnosis or treatment initiation as a starting point.

We found only a few studies that reported on the full cascade of care for LTBI published after 2005. From 2001 through the present, two large CDC-funded research consortia (the TB Trials Consortium and the TB Epidemiologic Studies Consortium) made up of many state and local TB programs have been conducting studies on LTBI diagnostics and treatment, which might have limited publications during the period while awaiting study results. [46]. Further, all studies were conducted before the CDC recommendation of the 3HP drug regimen (3 months of weekly doses of combined isoniazid and rifampin) that is much shorter and easier to complete than 6–9 months of daily or bi-weekly directly observed Isoniazid regimens [47].

Our review’s findings could be biased. Publication bias may create a non-representative sample: some program data, especially from smaller or poorly implemented efforts, might not be published in conventional scientific outlets or published at all. Collecting nationally generalizable data would require an extensive survey of existing programs, which was outside of the scope of this review. Measurement bias and detection bias are also a likely factor in the rates of LTBI-positive results. All but one study used TST for LTBI testing. TST interpretation is subjective, requiring measurement of skin induration (classified as ≥5, ≥10, or ≥15 millimeters) and interpretation in light of immune status (e.g., HIV infection) and history of TB exposure [48]. There can be both false positives (e.g., because of previous BCG vaccination that is common in FBP) and false negatives (e.g., because of cutaneous anergy).

Another potential limitation is that some studies could have intensively tracked participants, more than would be possible in routine TB programs, to minimize attrition. Although we collected such data if reported, the vast majority of studies did not provide detailed information pertaining to program implementation, thus making it infeasible to assess the potential impact of such activities. Finally, since the majority of participants were migrant farmworkers and could have moved before the completion of treatment, programs may have underestimated treatment outcomes by not considering treatment delivered by other providers.

Despite these limitations, our review provides the most comprehensive and up-to-date evidence-base for better understanding of the community-based TTT program performance in FBP in the United States. Our search strategies were comprehensive and highly sensitive, and used advanced techniques. We believe that we captured all potentially relevant studies indexed in several major bibliographic databases. We also searched grey literature, contacted study investigators and other experts in an effort to capture unpublished data. Although our findings are less applicable to community-based TTT programs that will use IGRA for testing (with improved performance compared to TST) and 3HP for treatment (shorter and easier to complete than 6–9 months of isoniazid), there are still programs using older test and treatment options. Further, new programs can estimate how much improvement they can expect by switching to IGRA because approximately 13% of persons in predominantly FBP groups who received TST never returned for the reading. Finally, our findings inform standard of care performance for comparative effectiveness and cost-effectiveness modeling for newer LTBI test and treatment options.

Our findings highlight a pressing need to develop a better understanding of barriers to retaining FBP in programs once they are reached. It is a waste of scarce resources to recruit FBP for testing but then manage to treat only a fraction of those testing positive. There is also an urgent need for strategies to increase testing and treatment uptake in specific FBP, particularly those originating in countries greatly contributing to the United States TB burden, such as Philippines, India, Vietnam, China, and Mexico.

## Supporting information

S1 File Systematic review protocol. (PDF)

S2 File PRISMA checklist. (PDF)

S3 File Database search strategies. (PDF)

S4 File Expanded extracted data. (XLSX)

S5 File Articles screened at full text level. (PDF)

S6 File Proportions in matrix format. (PDF)

S7 File Migrant farmworker subanalysis. (PDF)
