# Supplementary material for: Yield of community-based tuberculosis targeted testing and treatment in foreign-born populations in the United States: A systematic review
Source: PLoS One. 2017 Aug 7;12(8):e0180707. doi: 10.1371/journal.pone.0180707 (PMC5546677; doi:10.1371/journal.pone.0180707)
Supplement: S1 File — (PDF) [file pone.0180707.s001.pdf]

Study Title: Community-based strategies to identify and recruit foreign-born populations for latent tuberculosis infection screening in the United States: Protocol for a systematic review

Project: Cross-cutting Hard-to-reach Population

Consortium for the Assessment of Prevention Economics

UCSF: *Mohsen Malekinejad (Project lead & corresponding author), James G. Kahn (PI), Adithya Cattamanchi (co-investigator)***,** *Hacsi Horvath (Database search lead & lead author), Amanda Viitanen (data extraction lead & co-author), Devon McCabe (project manager)*

Centers for Disease Control and Prevention (CDC), Division of Tuberculosis Elimination (DTBE)
Suzanne Marks, Andrew Hill

***Correspondence:**

Mohsen Malekinejad, MD, DrPH, Institute for Health Policy Studies and Global Health Sciences, University of California San Francisco, 3333 California Street, Suite 265, San Francisco, CA 94118, USA. E-mail address: MMalekinejad@ucsf.edu. Phone: +1 (510) 8168555; Fax: +1 (415) 4760705

This project is supported by Cooperative Agreement (Grant No: U38PS004649) from the US Centers for Disease Control and Prevention (CDC)

University of California San Francisco

Table of Contents

ACKNOWLEDGEMENTS **1**

ABBREVIATIONS **2**

BACKGROUND **3**

OBJECTIVES **4**

METHODS **4**

- INCLUSION AND EXCLUSION CRITERIA: 4
- PICO FRAMEWORK 5
- SEARCH METHODS FOR IDENTIFYING STUDIES 7
- SCREENING AND DATA COLLECTION 8
- DATA EXTRACTION AND MANAGEMENT: 8
- RISK OF BIAS ASSESSMENT 9
- ANALYSIS 10

REFERENCES **12**

APPENDIX 1 .... **14**

- PUBMED SEARCH STRATEGY, WHICH WILL BE MODIFIED AND ADAPTED AS NEEDED FOR USE IN THE OTHER DATABASES. 14
- ADDITIONAL HIV/AIDS SEARCH TERMS (PUBMED EXAMPLE) 15

ACKNOWLEDGEMENTS

We would like to thank our colleagues at the California Department of Public Health for their contributions to this project: Jennifer Flood, Peter Oh, Christy Pak, Lisa Pascopella, Barry Pennen and Neha Shah.

ABBREVIATIONS

APHA	American Public Health Association
BCG	Bacillus Calmette-Guérin
CBA	Controlled before-and-after (trials)
IGRA	Interferon-gamma (IFN-c) release assays
LTBI	Latent tuberculosis infection
MeSH	Medical Subject Heading
Non-RCT	Non-randomized controlled trial
PICO	Population, intervention, comparator and outcome
RCT	Randomized controlled trial
RDS	Respondent-driven sampling
RR	Risk ratio
TST	Tuberculin skin test
TB	Tuberculosis

BACKGROUND

Worldwide, around 1.5 million people die of tuberculosis (TB) infection each year. [1] Nearly a third of people worldwide have latent TB infection (LTBI), which place them at increased risk of developing active TB. In otherwise-healthy people with LTBI, this risk is around 10%. [1] However, people with LTBI who have compromised immune systems or are tobacco users are at a much higher risk of developing active TB. [1]

The distribution of LTBI prevalence is widely varied among countries, and to a great extent negatively correlates with country income.[2] In the United States (US), those at highest risk of LTBI, as well as of developing active TB, are people who were born in less-wealthy countries and later came to the US. [3] In 2014, 66.5% (n=6181) of 9,412 active TB cases in the US occurred in foreign-born patients. [3] Newly diagnosed and reactivated TB infection among foreign-born (FB) individuals in the US is currently 13.4 times greater than among US-born persons (15.3 vs. 2.95 cases per 100 000 population, respectively). [3]

All immigrants seeking permanent residency or asylum in the United States go through routine screening for active TB. For those seeking asylum or permanent residency, pre-arrival screening is performed in the country of origin by a Panel Physician. [4, 5] B-waiver immigrants, who are screened for active TB internationally and have abnormal chest radiography are required to visit the health department after immigration for follow-up and treatment if necessary. For immigrants already in the US seeking a status adjustment, screening is performed by a civic surgeon. LTBI screening (via Mantoux TST) is mandatory for immigrants seeking a status adjustment. [4, 5]

"Undocumented" foreign-borne (FB) population (i.e. those in the US without legal status) and certain none immigrant visa holders (e.g., students, skilled workers) bypass this routine active TB screening process on entry. Undocumented FB population in particular are at high risk because they often delay accessing treatment until becoming acutely ill due to fear of deportation or lack of health care coverage, [6] Therefore, preventing progression to active TB by identifying and treating LTBI cases in "undocumented" populations, is essential to eliminating TB infection within the US. [7]

Generally, there are two ways that individuals with LTBI and residing in the US are identified and reported: "passive" and "active" methods.

In respect to "passive" case finding, individuals with suspected LTBI, based on a positive TST or positive Interferon-gamma (IFN-c) release assays (IGRAs), are referred to State or county health department TB clinics through referrals from healthcare providers for further evaluation and treatment. TST is the most widely used method of tuberculosis testing and is considered a reasonably accurate assessment of LTBI status in immunocompetent adults. [8] It is also the most widely used method of screening for those who previously received the Bacillus Calmette-Guérin (BCG) vaccine. BCG vaccine is not widely administered in the US but is part of routine childhood immunization in countries with a higher burden of TB (including countries of origin for many undocumented migrants). [9] This "passive" strategy is very limited, however, because most providers don't routinely screen foreign-born patients and report cases testing positive. High-risk foreign-born individuals don't routinely access the US health care system, much less request LTBI screening. The challenges in identifying LTBI among undocumented foreign-born populations are even more intense, given the frequent language barriers [10], transportation issues, work schedules[11] and limited economic resources in these communities. Additionally, undocumented

migrants are often geographically and culturally isolated and have poor health seeking behavior [6], especially if they have low self-perception of tuberculosis risk. [12]

There are also several "active" case finding approaches that have been effective in increasing LTBI screening in populations residing in the US. These have included contact investigation, workplace screening [13]; screening for entry into medication-assisted therapy and drug treatment programs [14]; refugee and naturalization programs [15, 16] and mandatory screening in the criminal justice system. [7, 17]

However, innovative strategies are needed to target foreign-born individuals who are not reached by these strategies and are not integrated into mainstream healthcare system. [11] The potential yield of these strategies is the focus of this systematic review.

Successful identification of "hidden" foreign-born populations, including undocumented migrants and immigrants, is critical to targeted TB testing and elimination efforts. [10] Strategies for identifying these populations can require special effort, as groups may be isolated not only from the general population but from each other. Carefully designed community-based strategies (e.g., venue-based, peer-referral, or social marketing) may reach certain hidden foreign-born subpopulations that wouldn't be reached using "passive" case finding methods. However, it is unclear which methods (and under what circumstances they are used) may yield higher efficiency (i.e., faster and less expensive recruitment).

This systematic review's objectives are to identify the most effective and efficient community-based strategies to recruit foreign-born populations at high risk of TB, in particular undocumented immigrants, to support the modeling of TB test-and-treat strategies and TB elimination.

OBJECTIVES

To inform our modeling of targeted LTBI testing and treatment programs in the US, we will systematically review the scientific literature to assess the effectiveness of community-based strategies to identify, test for LTBI, and link to treatment foreign-born populations in the US. Our primary focus is strategies to reach populations in the US without documented legal status.

We are also examining the cost of these strategies; these methods are addressed in a separate protocol.

METHODS

Overview

Our goal is to estimate the yield & efficiency of test and link strategies, not to compare different strategies. This is distinct from a typical systematic review, which focuses on effect sizes of interventions compared to a standard of care. As a result, our inclusion, search, and extraction strategies differ from a typical systematic review. Our inclusion criteria and searches are broader, and our extraction focuses on results achieved compared to having no strategy in place.

Inclusion and exclusion criteria

Any study presenting a community-based strategy that aims to reach foreign-born populations in the US for LTBI screening. We do not anticipate finding too many studies exclusively conducted among FB population. Thus in order to find sufficient LTBI screening studies for a meaningful synthesis, we will include US studies exploring strategies to recruit individuals in migrant populations (US-born or FB) as

long as studies have reported data for FB population as a sub-analysis or with clear indication on what portion of the population is FB

The following study designs will be eligible for inclusion

- Randomized controlled trials (RCTs)
- Non-randomized controlled trials (non-RCTs)
- Controlled before-and-after (CBA) studies
- Observational cohort studies (single or double arm)
- Case-control studies
- Cross-sectional studies

Criteria for exclusion

- Case reports, case series and studies that are primarily qualitative in nature.
- Studies which focus on diagnostic test accuracy, without considering program yield.

PICO framework

We use the population, intervention, comparator and outcome (PICO) [18] schema to outline our inclusion and exclusion criteria. As approaches to bringing foreign-born populations into care can more readily be described as “strategies” than “interventions,” we will substitute the former term in articulating our plan.

Population

The following populations will be eligible for inclusion

- Adult (18 and above) foreign-born populations (i.e., individuals who were born outside the US and arrived at any age in the US) and at the time of study are identified in community settings (homes, community centers, camps, streets, churches, social events, etc.). Our primary interest is in adults without documented immigration status in the US, and if we find enough data for this population, we will analyze them separately . Our searches will capture all studies in populations with legal immigration status in the US.
- Studies conducted among target populations who are defined by their ethnicity and with the evidence of being at high risk of LTBI (e.g., Asian, Hispanic) are provisionally included as long as there is a clear indication of what % of population was FB in order to identify FB specific data.

Criteria for exclusion

- Populations with a pre-existing roster (i.e., list of names with identifying demographic and contact information that can be used to relatively easily identify and contact FB population). This includes (but not limited to) populations who are identified in:
    - hospitals

- in jails, prisons, detention centers, and other facilities where participants are detained involuntarily
- School

Strategies

Definition: community-based strategies are those in which investigators/ program personnel (recruiters) do not have access to a roster from the target population (potential recruits) and the first contact (to recruit them for LTBI screening) is made in a community setting.

The following strategies will be eligible for inclusion:

- to increase the likelihood of identifying foreign-born populations at high risk of LTBI.
- to expedite the recruitment process of foreign-born populations
- to increase the likelihood of TB testing in foreign-born populations
- to expedite the TB testing process of foreign-born populations
- to reduce cost associated with the above activities

Criteria for exclusion

- Strategies focusing on activities after TB testing, including retention in treatment and care
- Strategies in which the initial point of contact occurred in a hospital, jail, or other facility as described above
- Strategies in which foreign-born populations are screened for LTBI before or during the process of entering the US

Comparator

Studies with or without comparators are eligible for inclusion.

Outcomes

Primary outcomes:

- Number of individuals who were reached
- Number of subjects reached and became eligible
- Number of eligible subjects consented for testing
- Number of eligible subjects who were tested
- Number of eligible whose test was read
- Number of eligible subjects who tested positive
- Number of eligible subjects who agreed to start treatment

- Number of eligible subjects who started treatment
- Any reported cost associated with steps above
- Any reported time interval (duration) associated with above

Secondary outcomes:

- Any reported barriers and facilitators

Search methods for identifying studies

We will conduct a comprehensive and exhaustive search of multiple bibliographic databases in an effort to identify all relevant studies of strategies to increase LTBI screening in foreign-born populations in the United States, regardless of language or publication status (published, unpublished, in press and in progress).

Journal and trial databases:

We will search for relevant studies in the following databases from the earliest records to the search date:

- Cochrane Central Register of Controlled Trials
- PsycINFO
- PubMed
- SCOPUS
- Web of Science

We will use appropriate Medical Subject Heading (MeSH) terms and keywords to identify relevant studies. The search strategy will be iterative, in that references of included studies will be searched for additional references. All languages will be included.

See **Appendix 1** for our PubMed search strategy, which will be modified and adapted as needed for use in the other databases.

Conference databases:

We will search conference abstract archives of the American Public Health Association (APHA), the National TB Conference and the International Union Against Tuberculosis and Lung Disease for all available abstracts of systematic reviews presented at these conferences from 2000 through 2015.

Searching other resources:

In addition to searching electronic databases, we will contact individual researchers, experts working in the field and colleagues at CDC to learn of any relevant studies that may exist in the "grey literature," or that may be in preparation or in press.

We will search current and archived issues of CDC's "TB Notes" newsletter (archive access to be provided by CDC).[a]

[a] All current and archived issues of this quarterly newsletter are available online and accessible to the public: http://www.cdc.gov/tb/publications/newsletters/notes/default.htm

Screening and data collection

The methodology for screening and data collection will be based on the guidance of Cochrane Handbook of Systematic Reviews of Interventions. [19] One author will perform a broad first cut of all downloaded material from the electronic searches to exclude citations that are plainly irrelevant. Two authors will read the titles, abstracts and descriptor terms of the remaining downloaded citations to identify potentially eligible studies. We will obtain full text copies for all citations identified as potentially eligible, and two authors will independently inspect these to establish the relevance of the study according to the pre-specified inclusion criteria. Where there is uncertainty as to the eligibility of the record, we will obtain and examine the full review.

Methods for selection of studies:

Two authors will independently apply the inclusion criteria to the primary studies, and any differences arising will be resolved by discussion with a neutral arbiter. We will examine studies for relevance based on the objectives, design, types of participants and outcome measures.

HIV studies:

Because there are similarities between HIV testing and LTBI testing in terms of outreach to high-risk patients, we may later consider, if LTBI-specific data are too sparse, the applicability of HIV testing studies to our immediate LTBI concerns. In this event, we would examine search results obtained by adding an optional string of relevant HIV/AIDS terms to our current string of TB terms. After de-duplicating references, we would consider studies assessing strategies for increasing uptake of HIV testing in migrant populations.

Data extraction and management:

Two authors will independently extract data into a standardized, pre-piloted data extraction form. The following characteristics will be extracted from each included study:

Domain	Indicator (Examples)
**Citation**	PI/Author name /organization name, Publication year
**Overall methods**	Project broad category, Disease (s) area, Study type, Study design, Desired number of participants, Total recruited participants
**Characteristics of population & setting**	Country, State/Province, County/city, Study setting, Target population (s), Eligibility criteria
**Recruitment methods**	Sampling/contacting method, Type of recruitment site (s), Type of fixed site, Sampling/recruitment duration, Method used for consenting, Type of biological specimen and test, Strategies to improve participation, Incentive provided,
**Project outputs**	# target population; reached, eligible; recruited / refused, consented, tested, tested (+), start & complete treatment. Date recruitment started, Sampling/recruitment duration

Note: For a given data point with a mix of US-born and FB populations, we consider the sample as being FB if at least 80% of the sample is FB. If less then 80% if the composition of the subjects is FB, we still include that data point, but we will separately analyze the data.

Risk of bias assessment

Two review authors will independently assess risk of bias in each study. We will resolve any disagreement by discussion or by involving a neutral third party to adjudicate. We will develop summary figures to denote bias risk in each included study individually, as well as across all included studies.

Studies without comparators:

Given the nature of this systematic review (not on the assessment of the efficacy of a public health intervention), we anticipate that most relevant studies are cross-sectional in nature or one time screening and follow-up (single arm cohort) of target population. There is no scientific consensus on a single tool for assessing the risk of bias or methodological quality in cross-sectional studies and other studies without comparators. To the degree it may be appropriate in a given study, we will look in particular for the following methodological issues:

- Study sample not representative of target population
- Flawed measurement of outcome
- Incomplete or inadequately short follow-up
- Other potential threats to validity

Studies with comparators:

We do not anticipate identifying any RCTs, or even observational studies with comparators. For all included studies with a comparator or control condition, we will use the bias assessment tool described in the Cochrane Handbook. [19] The Cochrane approach assesses risk of bias in individual studies across six domains:

- Sequence generation (checking for selection bias)
- Allocation concealment (checking for selection bias)
- Blinding (checking for performance bias and detection bias)
- Incomplete outcome data (checking for possible attrition bias through withdrawals, dropouts, protocol deviations)
- Selective reporting
- Other forms of bias

While the first three domains would obviously be irrelevant to non-RCTs, we will formally note their absence in such studies. Our summary figures will then represent overall bias risk more accurately.

Additional bias assessment in observational studies with comparators:

Although more than 200 instruments have been identified [19-21], there is no scientific consensus on a single generic tool for assessing risk of bias or methodological quality in observational studies with comparators. [19-21] During our bias assessment with the Cochrane instrument (and particularly during

our assessment of "other forms of bias") we will make note of any additional methodological issues that would likely increase bias risk. We will look in particular for the following:

- Failure to develop and apply appropriate eligibility criteria (comparability of groups)
- Flawed measurement of exposure and/or outcome
- Failure to adequately control confounding
- Incomplete or inadequately short follow-up
- Other potential threats to validity

Analysis

As appropriate, depending on study design, we will calculate and present recruitment rate ratio for studies with comparator group, and for studies without comparators, we will present reported adjusted and unadjusted numerators and denominators.

We do not anticipate identifying enough high quality data points that would allow us to conduct a formal meta-analysis. If possible, we will calculate summary statistics using meta-analytic methods. Where meta-analysis is not possible or is inappropriate, we will perform a narrative synthesis of results.

As needed, we will use the Review Manager 5 software (RevMan v 5.3, 2014) provided by the Cochrane Collaboration for statistical analysis [22] or Stata v 13.

Unit of analysis issues:

The unit of analysis will be the individual patient.

Dealing with missing data:

We will contact study authors if it is necessary to obtain data missing from published reports. If necessary and appropriate, we may impute data.

Assessment of heterogeneity:

We will use the I^2 and the τ^2 statistics to measure heterogeneity among included studies in each analysis. We anticipate substantial heterogeneity across studies, and thus meta-analysis of included studies will be undertaken with caution, if at all. For studies that are homogenous with respect to types of populations, the interventions that are compared, and outcome measures, we will calculate pooled risk ratios (RR). If indeed we find substantial heterogeneity, we will explore it by pre-specified subgroup analysis. If heterogeneity persists, we will perform sensitivity analyses, present results separately and propose explanations for the observed heterogeneity.

Assessment of reporting biases:

Where we suspect reporting bias we will attempt to contact study authors and ask them to provide missing outcome data. Where this is not possible, and the missing data are thought to introduce serious bias, we will explore the impact of including such studies in the overall assessment of results by a sensitivity analysis.

If any meta-analysis in our review includes 10 more studies, we will assess the potential for publication bias for the studies using a funnel plot. [19, 23] We will attempt to minimize the potential for

publication bias through rigorous review methods and by using comprehensive search strategies, including evaluating published and unpublished literature in all languages.

Data synthesis:

If appropriate, we will conduct meta-analysis, using Cochrane's Review Manager software (RevMan 2014). [22] Since we expect significant heterogeneity between or among studies given the diversity in populations and study settings, we will use a random effects model. If meta-analysis is not possible, a narrative synthesis of studies will be undertaken.

Subgroup analysis and investigation of heterogeneity:

In pooled results with high heterogeneity, we will explore heterogeneity through subgroup analyses of the following:

- HIV infection
- Other co-morbidities of LTBI concern (individually)
- Country of origin
- Urban or rural setting
- Region of the United States
- Timeframe of studies

Sensitivity analysis:

Where relevant, we will conduct sensitivity analysis to investigate the effect of excluding studies with high risk of bias, studies with arbitrary inclusion criteria etc.

Declarations of conflict of interest:

None known.

REFERENCES

1. World Health Organization (WHO). Tuberculosis Fact Sheet No. 104 Geneva, Switzerland 2015 [8 April 2015]. Available from: http://www.who.int/mediacentre/factsheets/fs104/en/.
2. Rieder HL. Epidemiologic basis of tuberculosis control: International Union Against Tuberculosis and Lung Disease (IUATLD); 1999.
3. Scott C, Kirking H, Jeffries C, Price S, Pratt R. Tuberculosis Trends--United States, 2014. Morbidity and Mortality Weekly Report (MMWR). 2015;64(10):265-9.
4. FAQs: Tuberculosis Component of the Technical Instructions (TIs) for the Medical Examination of Aliens in the United States.
5. Pareek M, Baussano I, Abubakar I, Dye C, Lalvani A. Evaluation of immigrant tuberculosis screening in industrialized countries. Emerg Infect Dis. 2012;18(9):1422-9. Epub 2012/08/31.
6. Vargas Bustamante A, Fang H, Garza J, Carter-Pokras O, Wallace SP, Rizzo JA, et al. Variations in healthcare access and utilization among Mexican immigrants: the role of documentation status. J Immigr Minor Health. 2012;14(1):146-55. Epub 2010/10/26.
7. Morano JP, Zelenev A, Walton MR, Bruce RD, Altice FL. Latent tuberculosis infection screening in foreign-born populations: a successful mobile clinic outreach model. Am J Public Health. 2014;104(8):1508-15. Epub 2014/06/13.
8. American Thoracic Society, Centers for Disease Control and Prevention. Targeted tuberculin testing and treatment of latent tuberculosis infection. Am J Respir Crit Care Med. 2000;161(4pt2):S221–S47.
9. Ritz N, Curtis N. Mapping the global use of different BCG vaccine strains. Tuberculosis (Edinb). 2009;89(4):248-51. Epub 2009/06/23.
10. Leng JF, Changrani J, Gany F. Language Discordance and Testing for Latent Tuberculosis Infection Among Recent Asian and Latino Immigrants. J Community Health. 2011;36(2):228-30.
11. Gany FM, Trinh-Shevrin C, Changrani J. Drive-by Readings: A Creative Strategy for Tuberculosis Control Among Immigrants. American Journal of Public Health. 2005;95(1):117-9.
12. El-Hamad I, Casalini C, Matteelli A, Casari S, Bugiani M, Caputo M, et al. Screening for tuberculosis and latent tuberculosis infection among undocumented immigrants at an unspecialised health service unit. International Journal of Tuberculosis and Lung Disease. 2001;5(8):712-6.
13. Gany FM, Trinh-Shevrin C, Changrani J. Drive-by readings: a creative strategy for tuberculosis control among immigrants. Am J Public Health. 2005;95(1):117-9. Epub 2004/12/30.
14. Schwarz RK, Bruce RD, Ball SA, Herme M, Altice FL. Comparison of tuberculin skin testing reactivity in opioid-dependent patients seeking treatment with methadone versus buprenorphine: policy implications for tuberculosis screening. Am J Drug Alcohol Abuse. 2009;35(6):439-44. Epub 2009/12/18.
15. Center for Disease Control and Prevention. CDC immigration requirements: technical instructions for tuberculosis screening and treatment using cultures and directly observed therapy. U.S. Department of Health and Human Services, Centers for Disease Control and Prevention, National Center for Emerging and Zoonotic Infectious Diseases, Division of Global Migration and Quarantine, 2009.
16. Posey DL, Naughton MP, Willacy EA, Russell M, Olson CK, Godwin CM, et al. Implementation of new TB screening requirements for U.S.-bound immigrants and refugees - 2007-2014. MMWR Morb Mortal Wkly Rep. 2014;63(11):234-6. Epub 2014/03/22.
17. Center for Disease Control and Prevention. Prevention and control of tuberculosis in correctional and detention facilities: recommendations from CDC. MMWR Recomm Rep. 2006;55(RR-9):1-44.

18. Richardson S, Wilson M, Nishikawa J, Hayward R. The well-built clinical question: a key to evidence-based decisions. ACP J Club. 1995;123(3):A12-3.
19. Cochrane Handbook for Systematic Reviews of Interventions. Higgins JPT, Green S, editors. West Sussex, England: John Wiley & Sons Ltd; 2008.
20. Viswanathan M, Berkman ND, Dryden DM, Hartling L. Assessing Risk of Bias and Confounding in Observational Studies of Interventions or Exposures: Further Development of the RTI Item Bank. Rockville, MD: Agency for Healthcare Research and Quality, 2013.
21. Deeks J, Dinnes J, D'Amico R, Sowden A, Sakarovitch C, Song F, et al. Evaluating non-randomised intervention studies. Health Technol Assess. 2003;7(27).
22. Review Manager (RevMan). v5.3 ed. Copenhagen: The Nordic Cochrane Center, The Cochrane Collaboration,; 2014.
23. Egger M, Smith GD, Schneider M, Minder C. Bias in meta-analysis detected by a simple, graphical test. Bmj. 1997;315(7109):629-34.

APPENDIX 1

PubMed search strategy, which will be modified and adapted as needed for use in the other databases.

Search	PubMed query	Items
**#8**	**#5 AND #6 AND #7**	**679**
#7	Search (United States[tiab] OR USA[tiab] OR US[tiab] OR Alabama[tiab] OR Alaska[tiab] OR Arizona[tiab] OR Arkansas[tiab] OR California[tiab] OR Colorado[tiab] OR Connecticut[tiab] OR Delaware[tiab] OR Florida[tiab] OR Georgia[tiab] OR Hawaii[tiab] OR Idaho[tiab] OR Illinois[tiab] OR Indiana[tiab] OR Iowa[tiab] OR Kansas[tiab] OR Kentucky[tiab] OR Louisiana[tiab] OR Maine[tiab] OR Maryland[tiab] OR Massachusetts[tiab] OR Michigan[tiab] OR Minnesota[tiab] OR Mississippi[tiab] OR Missouri[tiab] OR Montana[tiab] OR Nebraska[tiab] OR Nevada[tiab] OR New Hampshire[tiab] OR New Jersey[tiab] OR New Mexico[tiab] OR New York[tiab] OR North Carolina[tiab] OR North Dakota[tiab] OR Ohio[tiab] OR Oklahoma[tiab] OR Oregon[tiab] OR Pennsylvania[tiab] OR Rhode Island[tiab] OR South Carolina[tiab] OR South Dakota[tiab] OR Tennessee[tiab] OR Texas[tiab] OR Utah[tiab] OR Vermont[tiab] OR Virginia[tiab] OR Washington[tiab] OR West Virginia[tiab] OR Wisconsin[tiab] OR Wyoming[tiab] OR Central Valley[tiab] OR Los Angeles [tiab] OR San Francisco[tiab] OR Boston[tiab] OR Chicago[tiab] OR Dallas[tiab] OR Houston[tiab] OR NYC[tiab] OR Phoenix[tiab] OR Miami[tiab] OR Seattle[tiab] OR Fresno[tiab] OR Modesto[tiab] OR Madera[tiab]) OR (United States[MeSH] AND tuberculosis[tiab])	788905
#6	Search (migrant*[tiab] OR emigrant*[tiab] OR migratory[tiab] OR immigrant*[tiab] OR refugee*[tiab] OR bracero*[tiab] OR farm worker*[tiab] OR (farm*[tiab] AND work*[tiab]) OR (farm*[tiab] AND labor*[tiab]) OR farmworker*[tiab] OR agricultural worker*[tiab] OR laborer*[tiab] OR “construction workers”[tiab] OR “day workers”[tiab] OR promotora*[tiab] OR undocumented[tiab] OR indocumentado*[tiab] OR alien*[tiab] OR foreigner*[tiab] OR foreign-born[tiab] OR crops[tiab] OR orchards[tiab] OR border[tiab] OR ethnic*[tiab] OR Latino[tiab] OR Latina[tiab] OR Hispanic[tiab] OR Mexic*[tiab] OR Tijuana[tiab] OR Nogales[tiab] OR El Paso[tiab] OR Juarez[tiab] OR Latin America*[tiab] OR Central America*[tiab] OR Migrant Worker[MeSH] OR Migrant[MeSH] OR Emigrants and Immigrants[MeSH])	289171
#5	Search ((Tuberculosis[MeSH] OR tuberculosis[tiab] OR TB[tiab] OR tuberculin OR LTBI[tiab] OR "Latent Tuberculosis/diagnosis"[Mesh]) OR ("Contact Tracing"[Mesh] OR "contact tracing"[tiab] OR (contacts[tiab] AND trace[tiab]) OR (contacts[tiab] AND tracing[tiab]) OR "active case finding"[tiab] OR "case tracking"[tiab] OR (contact*[tiab] AND investigation[tiab])))	235218
#4	Search #1 OR #2 OR #3	3404148
#3	Search (outreach[tiab] OR mobile[tiab] OR recruit*[tiab] OR enrol*[tiab] OR (cultural*[tiab] AND relevan*[tiab]) OR community*[tiab] OR community-based OR participatory OR communities[tiab] OR participation[tiab] OR bilingual[tiab] OR bi-lingual[tiab] OR engage*[tiab] OR join*[tiab] OR (barrier*[tiab] AND	1218549

	facilitat*[tiab]) OR “hard to reach”[tiab] OR “difficult to reach”[tiab] OR “hard-to-reach”[tiab] OR “difficult-to-reach”[tiab] OR hidden population*[tiab])	
#2	Search (((Mass Screening[MeSH] OR screen*[tiab]) OR (Primary Health Care[MeSH] OR Culturally Competent Care[MeSH] OR Patient Acceptance of Health Care[MeSH] OR "Mobile Health Units"[Mesh] OR (primary[tiab] AND care[tiab]) AND (detect*[tiab] AND manage*[tiab]) OR (detect*[tiab] AND link*[tiab])) OR (Primary Health Care[MeSH] OR Culturally Competent Care[MeSH] OR Patient Acceptance of Health Care[MeSH] OR (primary[tiab] AND care[tiab]) AND (risk factor*[tiab] AND asymptomatic[tiab]) OR (risk factor*[tiab] AND latent[tiab])) OR "Tuberculin Test/methods"[Mesh] OR (TST[tiab] AND screen*[tiab])))	626126
#1	Search (Personnel Selection/methods[MeSH] OR Patient Participation[MeSH] OR Community-Based Participatory Research/organization & administration[MeSH] OR “Randomized Controlled Trial”[pt] OR controlled trial[tiab] OR clinical trial[tiab] OR randomized[tiab] OR randomised[tiab] OR randomly[tiab] OR random*[tiab] OR Observation[mh] OR observational*[tiab] OR cohort*[tiab] OR case-control*[tiab] OR cross-section*[tiab] OR longitud*[tiab] OR survey*[tiab] OR surveillance[tiab])	2029325

Additional HIV/AIDS search terms (PubMed example)

HIV/AIDS terms are shown in blue. These would be optional to the TB search terms.

((((Tuberculosis[MeSH] OR tuberculosis[tiab] OR TB[tiab] OR tuberculin OR LTBI[tiab] OR "Latent Tuberculosis/diagnosis"[Mesh]) OR ("Contact Tracing"[Mesh] OR "contact tracing"[tiab] OR (contacts[tiab] AND trace[tiab]) OR (contacts[tiab] AND tracing[tiab]) OR "active case finding"[tiab] OR "case tracking"[tiab] OR (contact*[tiab] AND investigation[tiab]))))) **OR** ((HIV*[tiab] OR HIV/AIDS[tiab] OR "human immunodeficiency"[tiab] OR "human immune deficiency"[tiab] OR "acquired immunodeficiency"[tiab] OR HIV Infection[MeSH]))
