# Supplementary material for: Yield of community-based tuberculosis targeted testing and treatment in foreign-born populations in the United States: A systematic review
Source: PLoS One. 2017 Aug 7;12(8):e0180707. doi: 10.1371/journal.pone.0180707 (PMC5546677; doi:10.1371/journal.pone.0180707)
Supplement: S3 File — (PDF) [file pone.0180707.s003.pdf]

Database Search Strategies (March 30, 2015)

PubMed

Search	PubMed query	Items
**#9**	**#6 AND #7 AND #8**	**679**
#8	Search (United States[tiab] OR USA[tiab] OR US[tiab] OR Alabama[tiab] OR Alaska[tiab] OR Arizona[tiab] OR Arkansas[tiab] OR California[tiab] OR Colorado[tiab] OR Connecticut[tiab] OR Delaware[tiab] OR Florida[tiab] OR Georgia[tiab] OR Hawaii[tiab] OR Idaho[tiab] OR Illinois[tiab] OR Indiana[tiab] OR Iowa[tiab] OR Kansas[tiab] OR Kentucky[tiab] OR Louisiana[tiab] OR Maine[tiab] OR Maryland[tiab] OR Massachusetts[tiab] OR Michigan[tiab] OR Minnesota[tiab] OR Mississippi[tiab] OR Missouri[tiab] OR Montana[tiab] OR Nebraska[tiab] OR Nevada[tiab] OR New Hampshire[tiab] OR New Jersey[tiab] OR New Mexico[tiab] OR New York[tiab] OR North Carolina[tiab] OR North Dakota[tiab] OR Ohio[tiab] OR Oklahoma[tiab] OR Oregon[tiab] OR Pennsylvania[tiab] OR Rhode Island[tiab] OR South Carolina[tiab] OR South Dakota[tiab] OR Tennessee[tiab] OR Texas[tiab] OR Utah[tiab] OR Vermont[tiab] OR Virginia[tiab] OR Washington[tiab] OR West Virginia[tiab] OR Wisconsin[tiab] OR Wyoming OR Central Valley[tiab] OR Los Angeles [tiab] OR San Francisco[tiab] OR Boston[tiab] OR Chicago[tiab] OR Dallas[tiab] OR Houston[tiab] OR NYC[tiab] OR Dallas[tiab] OR Houston[tiab] OR Phoenix[tiab] OR Miami[tiab] OR Seattle[tiab] OR Fresno[tiab] OR Modesto[tiab] OR Madera[tiab]) OR (United States[MeSH] AND tuberculosis[tiab])	788905
#7	Search (migrant*[tiab] OR emigrant*[tiab] OR migratory[tiab] OR immigrant*[tiab] OR refugee*[tiab] OR bracero*[tiab] OR farm worker*[tiab] OR (farm*[tiab] AND work*[tiab]) OR (farm*[tiab] AND labor*[tiab]) OR farmworker*[tiab] OR agricultural worker*[tiab] OR laborer*[tiab] OR “construction workers”[tiab] OR “day workers”[tiab] OR promotora*[tiab] OR undocumented[tiab] OR indocumentado*[tiab] OR alien*[tiab] OR foreigner*[tiab] OR foreign-born[tiab] OR crops[tiab] OR orchards[tiab] OR border[tiab] OR ethnic*[tiab] OR Latino[tiab] OR Latina[tiab] OR Hispanic[tiab] OR Mexic*[tiab] OR Tijuana[tiab] OR Nogales[tiab] OR El Paso[tiab] OR Juarez[tiab] OR Latin America*[tiab] OR Central America*[tiab] OR Migrant Worker[MeSH] OR Migrant[MeSH] OR Emigrants and Immigrants[MeSH])	289171
#6	Search ((Tuberculosis[MeSH] OR tuberculosis[tiab] OR TB[tiab] OR tuberculin OR LTBI[tiab] OR "Latent	235218

	Tuberculosis/diagnosis"[Mesh]) OR ("Contact Tracing"[Mesh] OR "contact tracing"[tiab] OR (contacts[tiab] AND trace[tiab]) OR (contacts[tiab] AND tracing[tiab]) OR "active case finding"[tiab] OR "case tracking"[tiab] OR (contact*[tiab] AND investigation[tiab])))	
#4	Search #1 OR #2 OR #3	3404148
#3	Search (outreach[tiab] OR mobile[tiab] OR recruit*[tiab] OR enrol*[tiab] OR (cultural*[tiab] AND relevan*[tiab]) OR community*[tiab] OR community-based OR participatory OR communities[tiab] OR participation[tiab] OR bilingual[tiab] OR bi-lingual[tiab] OR engage*[tiab] OR join*[tiab] OR (barrier*[tiab] AND facilitat*[tiab]) OR “hard to reach”[tiab] OR “difficult to reach”[tiab] OR “hard-to-reach”[tiab] OR “difficult-to-reach”[tiab] OR hidden population*[tiab])	1218549
#2	Search (((Mass Screening[MeSH] OR screen*[tiab]) OR (Primary Health Care[MeSH] OR Culturally Competent Care[MeSH] OR Patient Acceptance of Health Care[MeSH] OR "Mobile Health Units"[Mesh] OR (primary[tiab] AND care[tiab]) AND (detect*[tiab] AND manage*[tiab]) OR (detect*[tiab] AND link*[tiab])) OR (Primary Health Care[MeSH] OR Culturally Competent Care[MeSH] OR Patient Acceptance of Health Care[MeSH] OR (primary[tiab] AND care[tiab]) AND (risk factor*[tiab] AND asymptomatic[tiab]) OR (risk factor*[tiab] AND latent[tiab])) OR "Tuberculin Test/methods"[Mesh] OR (TST[tiab] AND screen*[tiab])))	626126
#1	Search (Personnel Selection/methods[MeSH] OR Patient Participation[MeSH] OR Community-Based Participatory Research/organization & administration[MeSH] OR “Randomized Controlled Trial”[pt] OR controlled trial[tiab] OR clinical trial[tiab] OR randomized[tiab] OR randomised[tiab] OR randomly[tiab] OR random*[tiab] OR Observation[mh] OR observational*[tiab] OR cohort*[tiab] OR case-control*[tiab] OR cross-section*[tiab] OR longitud*[tiab] OR survey*[tiab] OR surveillance[tiab])	2029325

SCOPUS (n=40)
(TITLE-ABS-KEY (united states OR usa OR us OR alabama OR alaska OR arizona OR arkansas OR california OR colorado OR connecticut OR delaware OR florida OR georgia) OR TITLE-ABS-KEY (hawaii OR idaho OR illinois OR indiana OR iowa OR kansas OR kentucky OR louisiana OR maine OR maryland OR massachusetts OR michigan OR minnesota OR mississippi OR missouri OR montana OR nebraska OR nevada OR new hampshire OR new jersey) OR TITLE-ABS-KEY (new mexico OR new york OR north carolina OR north dakota OR ohio OR oklahoma OR oregon OR pennsylvania) OR TITLE-ABS-KEY (rhode island OR south carolina OR south dakota OR tennessee OR texas OR

utah OR vermont OR virginia OR washington OR west virginia OR wisconsin OR wyoming OR central valley OR los angeles) OR TITLE-ABS-KEY (san francisco OR boston OR chicago OR dallas OR houston OR nyc OR dallas OR houston OR phoenix OR miami OR seattle OR fresno OR modesto OR madera)) **AND** ((TITLE-ABS-KEY (screen* OR (risk factor* AND latent) OR (risk factor* AND asymptomatic) OR (detect* AND manage*) OR tst)) AND (TITLE-ABS-KEY (tuberculosis OR tb OR tuberculin OR ltbi OR "contact tracing" OR (contacts AND trace) OR (contacts AND tracing)) OR TITLE-ABS-KEY ("active case finding" OR "case tracking" OR (contact* AND investigation)))) **AND** (((TITLE-ABS-KEY (migrant* OR emigrant* OR migratory OR immigrant* OR refugee* OR bracero* OR farm worker* OR farmworker*) OR TITLE-ABS-KEY (agricultural worker* OR laborer* OR promotora* OR undocumented OR indocumentado* OR alien* OR foreigner* OR foreign-born OR crops OR orchards) OR TITLE-ABS-KEY (border OR ethnic* OR latino OR latina OR hispanic OR mexic* OR tijuana OR nogales OR el paso OR juarez OR latin america* OR central america*)) OR (TITLE-ABS-KEY ((farm* AND work*) OR (farm* AND labor*)))) **AND** (TITLE-ABS-KEY (outreach OR mobile OR recruit* OR enrol* OR (cultural* AND relevan*) OR community*) OR TITLE-ABS-KEY (community-based OR participatory OR communities OR participation OR bilingual OR bi-lingual) OR TITLE-ABS-KEY (engage* OR join* OR (barrier* AND facilitat*) OR "hard to reach" OR "difficult to reach" OR "hard-to-reach" OR "difficult-to-reach" OR hidden population* OR TITLE-ABS-KEY=(Randomized Controlled Trial" OR controlled trial OR clinical trial randomized OR randomised OR randomly OR random* OR observational* OR cohort* OR case-control* OR cross-section* OR longitud* OR survey* OR surveillance)))

PsycINFO (n=26)
(tuberculosis OR TB OR tuberculin OR LTBI) AND (migrant* OR farmworker OR "farm worker" OR laborer* OR undocumented OR indocumentado OR mexico OR mexican* OR "latin american" OR "central american" OR guatemalan OR salvadoran OR latino OR latina OR hispanic) AND (screen* OR screening OR "case finding")

Web of Science (n=186)
TS= (united states OR usa OR us OR alabama OR alaska OR arizona OR arkansas OR california OR colorado OR connecticut OR delaware OR florida OR georgia) OR TS= (hawaii OR idaho OR illinois OR indiana OR iowa OR kansas OR kentucky OR louisiana OR maine OR maryland OR massachusetts OR michigan OR minnesota OR mississippi OR missouri OR montana OR nebraska OR nevada OR new hampshire OR new jersey) OR TS= (new mexico OR new york OR north carolina OR north dakota OR ohio OR oklahoma OR oregon OR pennsylvania) OR TS= (rhode island OR south carolina OR south dakota OR tennessee OR texas OR utah OR vermont OR virginia OR washington OR west virginia OR wisconsin OR wyoming OR central valley OR los angeles) OR TS= (san francisco OR boston OR chicago OR dallas OR houston OR nyc OR dallas OR houston OR phoenix OR miami OR seattle OR fresno OR modesto OR madera)) **AND** ((TS= (screen* OR (risk factor* AND latent) OR (risk factor* AND

asymptomatic) OR (detect* AND manage*) OR tst)) AND (TS= (tuberculosis OR tb OR tuberculin OR ltbi OR "contact tracing" OR (contacts AND trace) OR (contacts AND tracing)) OR TS= ("active case finding" OR "case tracking" OR (contact* AND investigation)))) **AND** (((TS= (migrant* OR emigrant* OR migratory OR immigrant* OR refugee* OR bracero* OR farm worker* OR farmworker*) OR TS= (agricultural worker* OR laborer* OR promotora* OR undocumented OR indocumentado* OR alien* OR foreigner* OR foreign-born OR crops OR orchards) OR TS= (border OR ethnic* OR latino OR latina OR hispanic OR mexic* OR tijuana OR nogales OR el paso OR juarez OR latin america* OR central america*)) OR (TS= ((farm* AND work*) OR (farm* AND labor*)))) **AND** (TS= (outreach OR mobile OR recruit* OR enrol* OR (cultural* AND relevan*) OR community*) OR TS= (community-based OR participatory OR communities OR participation OR bilingual OR bi-lingual) OR TS= (engage* OR join* OR (barrier* AND facilitat*) OR "hard to reach" OR "difficult to reach" OR "hard-to-reach" OR "difficult-to-reach" OR hidden population*))

Cochrane Central Register of Controlled Trials (n=1)
(tuberculosis OR TB OR tuberculin OR LTBI) AND (migrant* OR farmworker OR "farm worker" OR laborer* OR undocumented OR indocumentado OR mexico OR mexican* OR "latin american" OR "central american" OR guatemalan OR salvadoran OR latino OR latina OR hispanic) AND (screen* OR screening OR "case finding")

Grey lit search strategy

The systematic review methodologist performed a thorough grey literature search (substantively based on the search terms listed below) for key organization websites (using advanced Google syntax), relevant dissertations (ProQuest) and conference abstracts (American Public Health Association conference abstracts). He also used Google syntax to search for non-TB articles that might be useful. These searches yielded five dissertations, 2 conference abstracts, 3 non-TB peer reviewed articles and two key organization websites. Full text review of these documents and websites produced no additional data.

Primary search terms: (tuberculosis OR TB) AND (latent OR LTBI) AND (foreign-born OR undocumented)

Secondary search terms as needed to refine: control, screening, targeted, community, outreach
