# Supplementary material for: Yield of community-based tuberculosis targeted testing and treatment in foreign-born populations in the United States: A systematic review
Source: PLoS One. 2017 Aug 7;12(8):e0180707. doi: 10.1371/journal.pone.0180707 (PMC5546677; doi:10.1371/journal.pone.0180707)
Supplement: S5 File — (PDF) [file pone.0180707.s005.pdf]

Articles screened at the full text level

Systematic Review of Community-based latent tuberculosis infection (LTBI) screening to reach foreign-born individuals in the United States: UCSF CAPE Project

Contents

Included (N=15) ... 1
Excluded: Facility/institution based (N=13) ... 3
Excluded: Lacked outcome of interest (N=6) ... 4
Excluded: Conducted outside of the US (N=2) ... 5
Excluded: Outbreak investigation (N=2) ... 5
Excluded: Didn't target foreign born (N=2) ... 5
Excluded: Sample largely children (N=1) ... 5
Excluded: No eligible study included (reviews and reports) (N=1) ... 5
Excluded: Duplicate data (N=1) ... 6

Included (N=15)

CDC. Epidemiologic Notes and Reports Tuberculosis among Migrant Farm workers. MMWR 1986;35(29):467-9.

D'Lugoff MI, Jones W, Kub J, Glass N, Thompson D, Brinkley-Laughon S, et al. Tuberculosis screening in an at-risk immigrant Hispanic population in Baltimore city: an academic health center/local health department partnership. Journal of cultural diversity. 2002;9(3):79-85.

Desale M, Bringardner P, Fitzgerald S, Page K, Shah M. Intensified Case-Finding for Latent Tuberculosis Infection Among the Baltimore City Hispanic Population. J Immigr Minor Health. 2013;15(4):680-5.

Frees N, Polkowski J, Farmer R, Akin R, Bankowski MJ, Neuman M, et al. HIV-Infection, Syphilis, and Tuberculosis Screening among Migrant Farm-Workers - Florida, 1992 (Reprinted from Mmwr, Vol 41, Pg 723-725, 1992). Jama-Journal of the American Medical Association. 1992;268(15):1999-2000.

Gany FM, Trinh-Shevrin C, Changrani J. Drive-by readings: a creative strategy for tuberculosis control among immigrants. Am J Public Health. 2005;95(1):117-9.

Garcia JG, Matheny Dresser KS, Zerr AD. Respiratory health of Hispanic migrant farm workers in Indiana. American journal of industrial medicine. 1996;29(1):23-32.

Jacobson ML, Mercer MA, Miller LK, Simpson TW. Tuberculosis risk among migrant farm workers on the Delmarva peninsula. Am J Public Health. 1987;77(1):29-32.

McCurdy SA, Arretz DS, Bates RO. Tuberculin reactivity among California Hispanic migrant farm workers. American journal of industrial medicine. 1997;32(6):600-5.

Mooney L. Suffolk County (New York) Targeted Tuberculosis Testing and Treatment Program Among the Foreign-born, 2000-2004. TB Notes. 2006;2006(2):10-3.

Perez-Stable EJ, Slutkin G, Paz EA, Hopewell PC. Tuberculin reactivity in United States and foreign-born Latinos: results of a community-based screening program. Am J Public Health. 1986;76(6):643-6.

Poss JE. Factors associated with participation by Mexican migrant farmworkers in a tuberculosis screening program. Nurs Res. 2000;49(1):20-8.

Poss JE, Rangel R. A tuberculosis screening and treatment program for migrant farmworker families. Journal of health care for the poor and underserved. 1997;8(2):133-40.

Simmons JD, Hull P, Rogers E, Hart R. Tuberculosis control migrant study of 1988. N C Med J. 1989;50(6):309-10.

Trapé-Cardoso M, Subaran S, Bracker A, Sapiain E, Gould B. Latent tuberculosis among Latino migrant farmworkers in Connecticut. Conn Med. 2008;72(7):405-9.

Wieland ML, Weis JA, Olney MW, Aleman M, Sullivan S, Millington K, et al. Screening for Tuberculosis at an Adult Education Center: Results of a Community-Based Participatory Process. Am J Public Health. 2011;101(7):1264-7.

Excluded: Facility/institution based (N=13)

Case Study 2: Community-based service delivery to the foreign-born: San Francisco. Treating Latent TB Infection in High Risk Populations. San Franisco: UCF Curry TB Center.

Bauer S. Community clinic offers access to care. A system and a city collaborate to care for an immigrant population. Health progress (Saint Louis, Mo). 1993;74(8):42-4, 65.

Catlos EK, Cantwell MF, Bhatia C, Gedin S, Lewis J, Mohle-Boetani JC. Public health interventions to encourage TB class A/B1/B2 immigrants to present for TB screening. American Journal of Respiratory and Critical Care Medicine. 1998;158(4):1037-41.

Hass MR. Health seeking and patient adherence: Tuberculosis screening and Latino immigrants. . Ann Arbor, Michigan: University of California, Irvine, 1993.

Kowatsch-Beyer K, Norris-Turner A, Love R, Denkowski P, Wang SH. Utilization of a latent tuberculosis infection referral system by newly resettled refugees in central Ohio. The international journal of tuberculosis and lung disease : the official journal of the International Union against Tuberculosis and Lung Disease. 2013;17(3):320-5.

Morales-Meye G. Evaluation of latent tuberculosis infection screening in the field. Southern California CSU DNP Consortium, 2015.

Nelson ME, Fingar AR. Tuberculosis Screening and Prevention for Foreign-Born Students - 8 Years Experience at Ohio-University. Am J Prev Med. 1995;11(3):48-54.

Nelson R. Screening and treating tuberculosis in immigrants to the USA. The Lancet Infectious diseases. 2014;14(6):454-5.

Norton D. Tuberculosis screening for international students. J Am Coll Health. 2000;48(4):187-9.

Oh PK. The demography of tuberculosis in California in a time of transition: In search of empirical evidence to guide public health agencies' efforts to target tuberculosis

screening in immigrant communities. Ann Arbor, Michigan: University of California, Berkeley, 2014.

Turner MO, Elwood RK. Tuberculosis screening for immigrants and refugees: diagnostic outcomes in the state of Hawaii. Am J Respir Crit Care Med. 1997;155(2):771.

Varkey P, Jerath AU, Bagniewski SM, Lesnick TG. The epidemiology of tuberculosis among primary refugee arrivals in Minnesota between 1997 and 2001. Journal of travel medicine. 2007;14(1):1-8.

Walters JK, Sullivan AD. Impact of Routine Quantiferon Testing on Latent Tuberculosis Diagnosis and Treatment in Refugees in Multnomah County, Oregon, November 2009-October 2012. Journal of immigrant and minority health / Center for Minority Public Health. 2015.

Excluded: Lacked outcome of interest (N=6)

Cain KP, Garman KN, Laserson KF, Ferrousier-Davis OP, Miranda AG, Wells CD, et al. Moving toward tuberculosis elimination: implementation of statewide targeted tuberculin testing in Tennessee. Am J Respir Crit Care Med. 2012;186(3):273-9.

Carillo L, Sparano A, Fernandez L, Hamsho-Diaz P. 244586 Development & Evaluation of new materials for the iVivir a Todo Pulmón! TB fotonovela series. American Public Health Association 139th Annual Meeting and Exposition; Washington, D.C.2011.

Chandrasekar E, Kaur R, Song S, Kim KE. A comparison of effectiveness of hepatitis B screening and linkage to care among foreign-born populations in clinical and nonclinical settings. J Multidiscip Healthc. 2015;8:1-9.

Hamsho-Diaz P, Fernandex L, Mangan J, Galindo S, Wegener D, Lauzardo M. 245951 A Participatory Approach to Develop and Evaluate Tuberculosis Educational Products for Spanish-Speaking Foreign-born Persons. American Public Health Association 139th Annual Meeting and Exposition; Washington, D.C.2011.

Perumalswami PV, Factor SH, Kapelusznik L, Friedman SL, Pan CQ, Chang C, et al. Hepatitis Outreach Network: a practical strategy for hepatitis screening with linkage to care in foreign-born communities. J Hepatol. 2013;58(5):890-7.

State of Georgia. Tuberculosis Program Evaluation Guidelines. In: Dept. of Public Health, Division of Health Protection, editors. 2012.

Excluded: Conducted outside of the US (N=2)

Greenaway C, Sandoe A, Vissandjee B, Kitai I, Gruner D, Wobeser W, et al. Tuberculosis: evidence review for newly arriving immigrants and refugees. Can Med Assoc J. 2011;183(12):E939-E51.

Senties R, Ramon Blancarte J. Program of tuberculosis conrol on the Mexico--United States frontier. Salud publica de Mexico. 1966;8(4):571-2.

Excluded: Outbreak investigation (N=2)

CDC, TB Elimination Division. TB Notes. 2010(3).

Kim DY, Ridzon R, Giles B, Mireles T. Pseudo-outbreak of tuberculosis in poultry plant workers, Sussex County, Delaware. J Occup Environ Med. 2002;44(12):1169-72.

Excluded: Didn't target foreign born (N=2)

Ciesielski SD, Seed JR, Esposito DH, Hunter N. The epidemiology of tuberculosis among North Carolina migrant farm workers. Jama. 1991;265(13):1715-9.

Morano JP, Zelenev A, Walton MR, Bruce RD, Altice FL. Latent Tuberculosis Infection Screening in Foreign-Born Populations: A Successful Mobile Clinic Outreach Model. Am J Public Health. 2014;104(8):1508-15.

Excluded: Sample largely children (N=1)

Denison AV, Pierce JR. Enrollment in English-as-a-second-language class as a predictor of tuberculosis infection in schoolchildren. Public Health Reports. 1996;111(5):428-30.

Excluded: No eligible study included (reviews and reports) (N=1)

Robotin MC, George J. Community-based hepatitis B screening: what works? Hepatol Int. 2014;8(4):478-92. doi: 10.1007/s12072-014-9562-4. PubMed PMID: 25298848; PMCID: PMC4182585.

Excluded: Duplicate data (N=1)

Poss JE. Factors associated with participation by Mexican and Guatemalan migrant farmworkers in a tuberculosis screening program. [Unpublished dissertation]. In press 1998.
