# Supplementary material for: Yield of community-based tuberculosis targeted testing and treatment in foreign-born populations in the United States: A systematic review
Source: PLoS One. 2017 Aug 7;12(8):e0180707. doi: 10.1371/journal.pone.0180707 (PMC5546677; doi:10.1371/journal.pone.0180707)
Supplement: S6 File — (PDF) [file pone.0180707.s006.pdf]

Proportion and 95% confidence interval for TB testing and treatment cascade: community-based programs among foreign-born populations in the US.

a. Predominantly (>90%) foreign-born populations

	Recruited	Test Placed	Valid Results	Test (+)	Received CXR*	Offered Treatment	Started Treatment	Completed Treatment
Reached	0.51 (0.42, 0.59)	0.49 (0.40, 0.58)	0.43 (0.34, 0.51)	0.12 (0.09, 0.15)	0.13 (0.11, 0.14)	0.04 (0.03, 0.06)	0.11 (0.10, 0.12)	0.03 (0.00, 0.09)
Recruited		0.91 (0.70, 1.00)	0.71 (0.58, 0.83)	0.21 (0.13, 0.30)	0.00 (0.00, 0.05)	0.08 (0.05, 0.11)	0.00 (0.00, 0.05)	0.05 (0.03, 0.08)
Test Placed			0.87 (0.76, 0.95)	0.29 (0.25, 0.34)	0.19 (0.11, 0.29)	0.08 (0.03, 0.16)	0.12 (0.05, 0.22)	0.08 (0.02, 0.16)
Valid Results				0.39 (0.29, 0.50)	0.24 (0.14, 0.35)	0.10 (0.03, 0.20)	0.15 (0.07, 0.25)	0.09 (0.03, 0.17)
Test (+)					0.66 (0.35, 0.91)	0.35 (0.06, 0.70)	0.40 (0.14, 0.70)	0.28 (0.08, 0.54)
Received CXR						0.50 (0.38, 0.62)	0.66 (0.44, 0.85)	0.43 (0.19, 0.69)
Offered treatment							1.00 (0.92, 1.00)	0.60 (0.45, 0.75)
Started treatment								0.69 (0.53, 0.83)

LEGEND	0-1 Study	2 Studies	3-4 Studies	> 4 Studies

Used random-effects meta-analysis modeling to pooled proportions if > 1 studies

*CXR = chest X-ray

b. Majority (50-90%) foreign-born populations

	Recruited	Test Placed	Valid Result	Test (+)	Received CXR*	Offered Treatment	Started Treatment	Finished Treatment
Reached	0.85 (0.59 - 0.99)	0.85 (0.59 - 0.99	0.66 (0.44 - 0.85)	0.20 0.12 - 0.30	0.10 (0.05 - 0.16)	0.08 (0.03 - 0.16)	0.02 (0.01 - 0.03)	0.01 (0.00 - 0.01)
Recruited		1.00 (1.00 - 1.00)	0.94 (0.83 - 0.99)	0.30 (0.26 - 0.33)	0.25 (0.21 - 0.30)	0.15 (0.13 - 0.18)	No Data	0.01 (0.00 - 0.02)
Test Placed			0.92 (0.84 - 0.97)	0.30 (0.28 - 0.32)	0.27 (0.25 - 0.28)	0.17 (0.14 - 0.20)	No Data	0.01 (0.00 - 0.02)
Valid Result				0.34 (0.29 - 0.39)	0.23 (0.15 - 0.32)	0.16 (0.10 - 0.22)	0.12 (0.00 - 0.35)	0.02 (0.01 - 0.03)
Test (+)					0.80 (0.53 - 0.97)	0.56 (0.46 - 0.66)	0.35 (0.03 - 0.79)	0.06 (0.03 - 0.11)
Received CXR						0.66 (0.53 - 0.77)	0.31 (0.22 - 0.42)	0.07 (0.03 - 0.12)
Offered Treatment							0.43 (0.26 - 0.63)	0.12 (0.05 - 0.21)
Started Treatment								0.90 (0.60 - 0.98)

LEGEND	0-1 Study	2 Studies	3-4 Studies	> 4 Studies

Used random-effects meta-analysis modeling to pooled proportions if > 1 studies

*CXR = chest X-ray
