# Supplementary material for: Yield of community-based tuberculosis targeted testing and treatment in foreign-born populations in the United States: A systematic review
Source: PLoS One. 2017 Aug 7;12(8):e0180707. doi: 10.1371/journal.pone.0180707 (PMC5546677; doi:10.1371/journal.pone.0180707)
Supplement: S7 File — (PDF) [file pone.0180707.s007.pdf]

Appendix S7: Proportion and 95% confidence interval for subset of steps in TB testing and treatment cascade: community-based programs among foreign-born populations in the US by target population and percent foreign-born

1 - Predominantly foreign-born population

Cascade steps	# Studies	N	Proportion	95% CI	Studies	N	Proportion	95% CI
	Migrant Farmworkers				Other Target Population			
Recruited / Reached	2	725	0.46	0.43 - 0.50	1	584	0.51	0.47 - 0.55
Test placed / Recruited	2	335	0.98	0.96 - 0.99	1	298	0.89	0.85 - 0.92
Test read / Test placed	3	465	0.92	0.58 - 1.00	5	2377	0.84	0.71 – 0.94
Test (+) / Test read	5	938	0.34	0.21 - 0.48	6	2940	0.43	0.28 – 0.59
CXR / Test (+)	1	15	0.00	0.00 - 0.20	3	672	0.79	0.46 – 0.99
Offered tx / CXR	2	101	0.72*	0.63 - 0.81	1	31	0.29	0.16 - 0.47
Offered tx / Test (+)	1	15	0.00	0.00 - 0.20	3	142	0.62	0.18 - 0.97
Start tx / Offered tx	1	30	0.3*	0.17 - 0.48	2	50	0.71	0.57 - 0.83
Finished tx / Start tx	1	9	0.89*	0.57 - 0.98	3	477	0.69	0.53 – 0.83

2 – Majority foreign-born population

Cascade steps	# Studies	N	Proportion	95% CI	Studies	N	Proportion	95% CI
	Migrant Farmworkers				Other Target Populations			
Recruited / Reached	3	1936	0.85	0.59-0.99	0	0	-	-
Test placed / Recruited	3	1598	1.00	1.00-1.00	0	0	-	-
Test read / Test placed	3	1598	0.92	0.84-0.97	1	2193	0.85	0.84-0.87
Test (+) / Test read	7	3632	0.34	0.28-0.41	1	1871	0.34	0.32-0.36
CXR / Test (+)	3	257	0.74	0.35-0.99	1	639	0.92	0.90-0.94
Offered tx / CXR	2	121	0.59	0.50-0.67	1	590	0.72	0.53-0.77
Offered tx / Test (+)	3	274	0.52	0.46-0.58	1	639	0.67	0.63-0.70
Start tx / Offered tx	1	23	0.43	0.26-0.63	0	0	-	-
Finished tx / Start tx	1	10	0.90	0.60-0.98	0	0	-	-

Tx = Treatment

X / Y = means proportion of “X” of those who proceeded to stage “Y”. For example, Recruited / Reached: proportion of recruited of those who were reached)
